# DNAmix 2021: Laboratory policies, procedures, and casework scenarios summary and dataset

**DOI:** 10.1016/j.dib.2023.109150

**Published:** 2023-04-14

**Authors:** Lauren M. Brinkac, Nicole Richetelli, Jonathon M. Davoren, Robert A. Bever, R. Austin Hicklin

**Affiliations:** a Noblis^1^, Reston, VA, USA; b Bode Technology^2^, Lorton, VA, USA

**Keywords:** Forensic science, DNA Mixtures, Probabilistic genotyping, Inter-laboratory variation, Policies and procedures, Casework scenarios

## Abstract

DNAmix 2021 is a large-scale study conducted to evaluate the extent of consistency and variation among forensic laboratories in the interpretation of DNA mixtures, and to assess the effects of various potential sources of variability. This study utilized a multi-phasic approach designed to collect information about participating laboratories, laboratory policies, and their standard operating procedures (SOPs). It also characterizes the degree of variation in assessments of suitability and number of contributors as well as in comparisons and statistical analyses of DNA mixture profiles. This paper specifically details the study design and the data collected in the first two phases of the study: the *Policies & Procedures (P&P) Questionnaire* and the *Casework Scenarios Questionnaire (CSQ)*. We report on the variation in policies and SOPs for 86 forensic laboratories—including information about their DNA workflows, systems, and type of statistics reported. We also provide details regarding various case-scenario specific decisions and the nature of mixture casework for 83 forensic laboratories. The data discussed in this article provide insight into the state of the field for forensic DNA mixture interpretation policies and SOPs at the time of the study (2021–2022).

**Specifications Table**

**Table:** 

Subject	Forensic science
Specific subject area	DNA mixtures
Type of data	Tables, graphs, text descriptions
How the data were acquired	Web-based questionnaires completed by laboratories that conduct DNA mixture casework
Data format	Raw, analyzed
Description of data collection	Forensic laboratories that conduct DNA mixture casework were asked to answer a variety of questions regarding their policies and procedures (e.g., DNA workflow, use of software, interpretation/ comparison/ statistical analysis details), decisions related to casework scenarios (e.g., case information provided, analysis options), and the nature of their mixture casework (e.g., types of samples, number of contributors).
Data source location	Data collected and archived by Noblis (Reston, VA, USA)
Data accessibility	Included within this article and in the OSF data archive “Laboratory Policies, Procedures, and Casework Scenario Decisions Relevant to DNA Mixture Interpretation: Data from the DNAmix 2021 Study” [1]. Repository name: OSF Data identification number: DOI:10.17605/OSF.IO/87SM4
	Direct URL to data: https://doi.org/10.17605/OSF.IO/87SM4

## Value of the Data

• Although there have been previous studies of interpretations of DNA mixtures (notably MIX13 [2], the STRmix Interlaboratory Study [3], and the DoD DFSC DNA Mixture Interpretation Study [4]; see review in [5]), there has not been a large-scale independent study evaluating the extent of variation in interpretation/statistical analysis of DNA mixtures:
  ○ Including results from state-of-the-practice probabilistic genotyping software (PGS)
  ○ With samples selected to be representative of the range of attributes found in actual DNA mixture casework
  ○ Using only real human DNA samples and no contrived or simulated profiles
  ○ Not restricted to specific products or statistical approaches
• The data included here may provide insight into the state of the field at the time of this study (2021–2022) in DNA mixture analysis, specifically regarding laboratory policies and procedures, case scenario decisions, and the nature of mixture casework.
• These responses characterize the level of inter- and intra-laboratory variation in reporting of laboratory standard operating procedures (SOPs). By extension, this data set provides greater visibility into various potential sources of variability in the interpretation, comparison, and statistical analysis of DNA mixtures.
• This data set may be of particular interest to laboratory managers and standards organizations, and may assist the community in deciding how to improve operational procedures, training, and standardization for DNA mixture analysis.
• The data can also be leveraged by other researchers and stakeholders to dig deeper into the extent of variation in DNA mixture policies, procedures, and decisions related to scenarios/case-specific factors—as well as to explore the interrelations between these factors.

## 1 Objective

This paper and associated data table [1] detail the study design and the data collected in the first two phases of the DNAmix 2021 study: the *Policies & Procedures (P&P) Questionnaire* and the *Casework Scenarios Questionnaire (CSQ)*. We report on the variation in policies and SOPs for 86 forensic laboratories—including information about their DNA workflows, systems, and type of statistics reported. We also provide details regarding various case-scenario specific decisions and the nature of mixture casework for 83 forensic laboratories. The data discussed in this article provide information regarding the state of the field at the time of this study for forensic DNA mixture interpretation policies and SOPs.

## 2 Data Description

This paper and its supporting tables present information describing the variation in policies and procedures and casework scenario decisions related to the interpretation of DNA mixtures. This information was collected in the first two phases of *DNAmix 2021*, which was a four-phased study of inter-laboratory variation in DNA mixture interpretation (see Section 5.1 for details).

“Laboratory Policies, Procedures, and Casework Scenario Decisions Relevant to DNA Mixture Interpretation: Data from the DNAmix 2021 Study” [1] (hereafter “the P&P-CSQ dataset”) is a spreadsheet that includes responses to the *Registration and Configuration Questionnaire* (RC), the *Policies & Procedures Questionnaire* (P&P; collected as Phase 1 of DNAmix 2021), and the *Casework Scenarios Questionnaire* (CSQ; collected as Phase 2 of DNAmix 2021). This paper presents the experimental design, materials and methods, and summaries of the information included in the P&P-CSQ dataset [1].

The summaries in this paper present results by laboratory: for laboratories with multiple participants (referred to as subunits), the majority response is used for each laboratory; if a majority is not available for a given question (due to multiple different subunit responses with no majority response), the responses for that laboratory were flagged as “Inconsistent” for that question. The P&P-CSQ dataset [1] includes the raw participant responses as well as the majority responses aggregated by laboratory; summary tables for each questionnaire are also included which provide the verbatim questions and answer responses as presented to participants. All responses have been de-identified in accordance with the study's informed consent agreement, which assured participants of confidentiality.

Supplemental material also includes the study instructions documents along with the Informed Consent Form and Frequently Asked Questions (FAQs) as Supplemental Data S1.

### 2.1 Overview

In order to characterize the state of the field for DNA mixture interpretation, the first two phases of DNAmix 2021 were designed to collect information about the policies and validated procedures in participating laboratories’ standard operating procedures (SOPs), as well as to characterize the nature of their mixture casework at the time of the survey.

The P&P-CSQ dataset [1] provides the raw responses collected in these first two phases of DNAmix2021 and summarizes the data in several manners (for ease of interpretation and use by stakeholders); the spreadsheet includes an informational README sheet and nine de-identified data sheets:

• RC by Participant (*n* = 190 participants): all responses to the RC questionnaire
• P&P by Participant (*n* = 178 participants): all responses to the P&P questionnaire (Phase 1 of DNAmix 2021)
• CSQ by Participant (*n* = 163 participants): all responses to the CSQ questionnaire (Phase 2 of DNAmix 2021)
• RC-subset by Lab (*n* = 86 labs): summary of registration responses by laboratory, limited to participants that completed the P&P questionnaire
• P&P by Lab (*n* = 86 labs): summary of P&P responses by laboratory
• CSQ by Lab (*n* = 83 labs): summary of CSQ responses by laboratory
• RC Questionnaire Summary (*n* = 86 labs; *n* = 178 participants): verbatim questions and answers from the RC questionnaire, with summary counts by lab and by participant
• P&P Questionnaire Summary (*n* = 86 labs; *n* = 178 participants): verbatim questions and answers from the P&P questionnaire, with summary counts by lab and by participant
• CSQ Questionnaire Summary (*n* = 83 labs; *n* = 163 participants): verbatim questions and answers from the CSQ questionnaire, with summary counts by lab and by participant

Table 1, Table 2, Table 3, Table 4, Table 5, Table 6, Table 7, Table 8, Table 9, Table 10, Table 11, Table 12, Table 13, Table 14, Table 15, Table 16, Table 17, Table 18, Table 19, Table 20, Table 21, Table 22, Table 23, Table 24, Table 25, Table 26, Table 27, Table 28, Table 29, Table 30 in the following sub-sections provide a summary of the response data included in the P&P-CSQ dataset [1].

### 2.2 Test Yield

A total of 179 participants from 87 forensic laboratories completed at least one phase of the study.^1^ Not all participants completed all phases of the study: 89 participants completed all four phases of the study, and the remaining 90 participants completed at least one phase. With respect to laboratories, 45 laboratories had at least 1 participant complete all four phases of the study, and an additional 42 laboratories had at least 1 participant complete at least one phase. The following are the total responses for each phase (out of the 179 participants from 87 different laboratories):

• *Phase 1: Policies & Procedures (P&P) Questionnaire* — 178 responses reported by 178 participants from 86 laboratories
• *Phase 2: Casework Scenarios Questionnaire (CSQ)* — 163 responses reported by 163 participants from 83 laboratories
• *Phase 3: Number of Contributors (NoC) Subtest* — 1507 responses reported by 134 participants from 67 laboratories
• *Phase 4: Interpretation, Comparison, and Statistical Analysis (ICSA) Subtest* — 765 responses reported by 106 participants from 52 laboratories

The following sections summarize the results for the first two phases, reported by the majority response for each laboratory. For all response data for these phases, including a breakdown by individual participant, see the P&P-CSQ dataset [1].

### 2.3 Study Population for the P&P and CSQ Studies

Table 1 shows the distribution of laboratory type by U.S./non-U.S. labs for the laboratories who completed the *P&P Questionnaire* and/or the *CS Questionnaire*. (The populations were largely the same, except for 4 labs that participated in *P&P* but not *CSQ*, and 1 lab that participated in *CSQ* but not *P&P*.) The majority of labs were U.S. labs; the non-U.S. labs represented at least 7 different countries other than the U.S.^2^ The U.S. State laboratories that participated in *P&P* represented 29 states (28 states for *CSQ*). Laboratories spanned a range of sizes—from 1 to 51+ DNA analysts. Participating labs most commonly employed a range of 2 to 25 analysts (RC#04).

**Table 1:** Distribution of types of forensic laboratories completing P&P and CSQ. (Question RC#03).

Category		# Labs (P&P Study)	# Labs (CSQ Study)
U.S. laboratories	Federal	2	1
	State	32	32
	Local	38	37
	Private	2	1
	*N/A: inconsistent*	1	1
	Subtotal	75	72
Non-U.S. laboratories	Federal/National	3	3
	State/Provincial	8	8
	Subtotal	11	11

Of the 86 laboratories that submitted responses to *P&P*, 61 had a single registered subunit and 25 had multiple registered subunits—9 labs had 2 subunits, 5 labs had 3 subunits, 3 labs had 4 or 7 subunits, and the remaining labs each had 5, 6, 11, 14, or 15 subunits. Of the 83 laboratories that submitted responses to *CSQ*, 60 had a single registered subunit and 23 had multiple registered subunits—10 labs had 2 subunits, 3 labs had 3 or 4 subunits, 2 labs had 5 or 7 subunits, and 1 lab had 11, 13, or 14 subunits.

### 2.4 Policies and Procedures (P&P) Questionnaire Responses

This section provides a summary and overview of the results from *Phase 1: Policies & Procedures Questionnaire* that are included in detail in the P&P-CSQ dataset [1]. This section also summarizes responses to a few questions from the *Registration and Configuration Questionnaire* (RC) that are relevant to *P&P*. Each section below indicates the specific question number being presented, prefixed with “RC#” (*RC Questionnaire*) or “PP#” (*P&P Questionnaire*).

Participants were instructed to answer all questions as completely and accurately as possible, based upon their laboratory's SOPs and any other policies and validated procedures utilized for DNA mixture casework.

As discussed in Section 4.2 (Test Yield), all results in this section are based on the responses from the 86 forensic laboratories that completed the *P&P Questionnaire*. Responses are reported by laboratory: for laboratories with multiple subunits, these results are based on the majority response among the subunits for each laboratory; labs without a majority response are flagged as “N/A: inconsistent.” Totals for all tables total 86 labs, except for questions that indicate “check all that apply,” which generally total more than 86.

#### 2.4.1 Quantification

Of the 86 participating labs, 50 use the Applied Biosystems Quantifiler™ Trio quantification kit and 20 used Promega Power Quant; no more than five labs used any other quantification kit (PP#02). All but four laboratories (*n* = 82) use the kit standard for DNA quantification (PP#03).

Table 2 outlines several DNA quantification factors used to determine the suitability of a sample to move forward to amplification. About three-quarters of laboratories terminate analysis prior to amplification depending upon the total DNA quantity. These thresholds vary widely— 6 labs list a threshold of 0 ng, 30 labs terminate analysis if there is 0.01 ng or less, and 44 labs terminate analysis if there is 0.05 ng or less (mean 0.0432 ng, median 0.0125 ng, range 0.0–0.24 ng). Just over half of the participating labs terminate analysis prior to amplification based upon the proportion of total male DNA (if the POI is male)—20 labs terminate analysis if the male fraction is 1% or less and 44 labs terminate analysis if the male fraction is 5% or less (mean 1.9%, median 1.7%, range 0.0–5.0%). In general, lab SOPs do not require an analyst to terminate analysis based upon degradation index (DI)—the single lab that does uses a DI=2 threshold.

**Table 2:** Suitability factors considered to determine whether to terminate analysis prior to amplification. See the P&P-CSQ dataset [1] for other factors.

Suitability factors		# Labs
Total DNA quantity (PP#04)	Yes	64
	No	22
Proportion total male DNA (PP#05)	Yes	47
	No	37
	*N/A: inconsistent*	2
Other factors (PP#06)	Yes	30
	No	55
	*N/A: inconsistent*	1
Degradation index (PP#07)	Yes, specified by SOPs	1
	No, but DI may influence target input	56
	No, DI is not considered when performing amplification	24
	Not specified by SOPs, left to analysts’ discretion	2
	*N/A: inconsistent*	3

#### 2.4.2 Amplification

Table 3 details the amplification kits and amplification cycle numbers used by participating laboratories, specifically for analysis of DNA mixture samples. Applied Biosystems GlobalFiler is the most commonly used amplification kit (run at 29 cycles, or less commonly at 28 cycles), followed by Promega Powerplex Fusion 6C (run at 29 cycles). These responses were used for the selection of amplification/capillary electrophoresis (Amp/CE) settings for subsequent phases of the study.

**Table 3:** DNA amplification kits and number of amplification cycles used by participating laboratories for DNA mixtures (*n* = 86 labs). “Check all that apply” responses may total greater than 86. See the P&P-CSQ dataset [1] for details about combinations of responses that were selected. (Questions RC#05-RC#06).

		Amplification cycles (check all that apply)								
Amplification kits (check all that apply)	# Labs	<24	24	25	26	27	28	29	30	>30
Applied Biosystems AmpFLSTR Identifiler	1						1			
Applied Biosystems AmpFLSTR Identifiler Plus	7					1	7	2	2	
Applied Biosystems AmpFLSTR Profiler	1						1			
Applied Biosystems AmpFLSTR Profiler Plus	1						1			
Applied Biosystems GlobalFiler	44					1	13	31	4	1
Promega Powerplex 16	1									1
Promega Powerplex 16 HS	1									1
Promega Powerplex Fusion 5C	9						2	5	2	
Promega Powerplex Fusion 6C	25			1		1	3	23	1	1
QIAGEN Investigator 24plex	6				1		1	2	2	
Other	4									

**Fig. 1:**
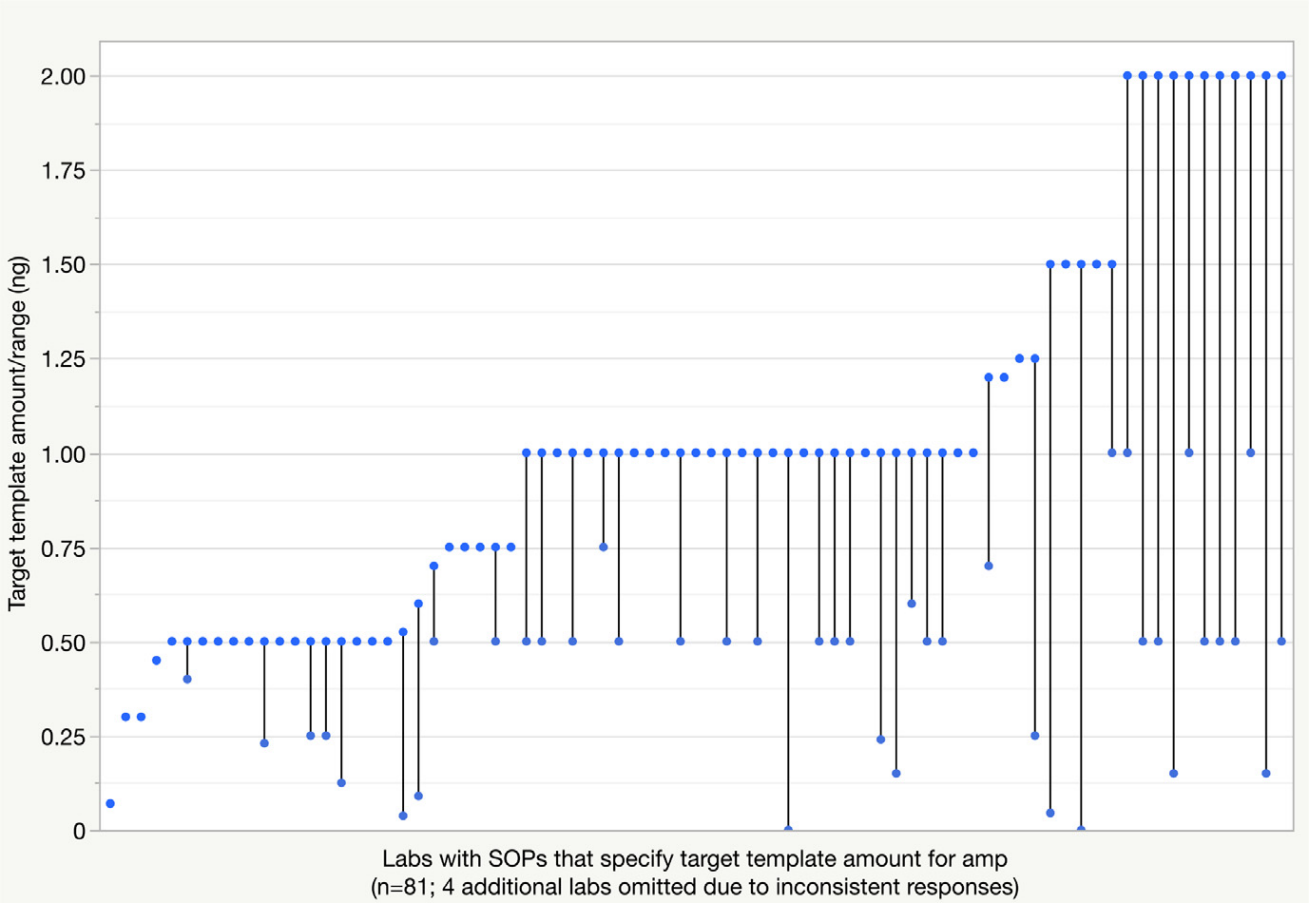
Target template amount of DNA used for amplification, as specified by the SOPs of participating laboratories. Points indicate a single target amount; lines indicate the lab specified a range. (PP#09).

With respect to amplification volume, the vast majority of participating labs use either a 25 µL (64 labs) or a 15 µL (13 labs) total amplification volume (PP#08). Note that since the amplification kit was a “check all that apply” question asked in *RC* and the amplification volume question was a free text entry asked in *P&P*, we do not have the data to reliably report the intersection between the two responses. Target DNA template varied notably (Fig. 1)—amounts ranged from 0 ng to 2 ng, with 42 labs specifying a target template range and 35 labs specifying a single target amount (mean 0.78 ng, std dev: 0.34 ng, median: 0.75 ng) (PP#09).

Just over half of participating laboratories (*n* = 45) use at least one type of enhanced amplification method (PP#10). Concentration of the sample was used by 38 labs, and 14 labs used increased injection time. No more than five labs used any other methods (increasing the volume of amplicon (in CE), increasing cycle number, decreasing the amplification volume, decreasing the amplicon volume, and post amplification cleanup). The majority of labs that reported using an enhanced amplification method (31/45), employ just a single method from the list of options.

Table 4 outlines policies and procedures related to the use of replicate amplifications for DNA mixture samples in casework. Just over one-quarter of laboratories routinely use replicate amplifications and when they do, they most often use all replicates in interpretation.

**Table 4:** Policies regarding the utilization of replicate amplifications. “Check all that apply” responses may total greater than 86. See the P&P-CSQ dataset [1] for details about combinations of responses that were selected.

Category		# Labs
Use replicate amplifications (PP#11)	Yes	24
	No	60
	N/A: inconsistent responses	**2**

Circumstances for utilizing replicate amplifications (check all that apply) (PP#12) *Limited to the 24 labs that use replicate amplifications*	We always amplify in duplicate/triplicate	3
	Based on limited/minimal DNA input amount	9
	Based on number of contributors	5
	Based on lowest contributor percentage	3
	Based on PGS diagnostics	3
	Based on lowest contributor average RFU	2
	Based on statistical value computed for POI	1
	Other	10

Incorporate all replicate amplifications in interpretation (PP#13) *Limited to the 24 labs that use replicate amplifications*	Yes, we use all replicate amplifications in interpretation	13
	Depends on the sample	5
	No, we select which (one) replicate amplification to use in interpretation	2
	Other	2
	N/A: inconsistent responses	**2**

#### 2.4.3 Capillary Electrophoresis (CE) Instrument

Tables 5 and 6 detail the CE instrument(s), injection voltage(s), and injection time(s) used by participating labs, specifically for analysis of DNA mixture samples.^3^ The ABI 3500 series instruments are used by the vast majority of laboratories and are generally run at 1.2 kV injection voltage. Injection time is much more variable than injection voltage, but 15 s for ABI 3500 and 24 s for ABI 3500xl are the most commonly used times. Note: these responses were used for the selection of Amp/CE settings for subsequent phases of the study. For additional details regarding injection, buffer, and ILS volumes, refer to the P&P-CSQ dataset [1], PP#16-18.

Eighty-three laboratories never change from their default/standard injection voltage and 61 labs never change from their default injection time. Of the 23 labs that do change the default/standard injection time, all but one report that it is at the analyst's discretion.

**Table 5:** CE instrument(s) and injection voltage(s) (kV) validated and used by participating laboratories for mixture casework. “Check all that apply” responses may total greater than 86. See the P&P-CSQ dataset [1] for details about combinations of responses that were selected. (Questions RC#07-RC#08).

CE Instrument		Injection voltage (kV) (check all that apply)						
(check all that apply)	# Labs	1.2	1.3	1.4	1.5	1.6	3	>3.0
ABI 3130	4	1					3	
ABI 3130xl	4	1					3	1
ABI 3500	50	47	1		1		1	
ABI 3500xl	38	35		1		2	1	1
Other	1

**Table 6:** CE instrument(s) and injection time(s) (seconds) validated and used by participating laboratories for mixture casework. See the P&P-CSQ dataset [1] for details about combinations of responses that were selected. (Question RC#09).

		Injection time(s) (check all that apply)																					

CE Instrument (check all that apply)	# Labs	<5	5	6	7	8	9	10	11	12	13	14	15	16	17	18	19	20	21	22	23	24	>24
ABI 3130	4	1	4		1		1	2															
ABI 3130xl	4	2	4		2			2															
ABI 3500	50	2	7	3	6	2	3	11	2	5	2	2	36	2	2	1	1	1	2	2	1	6	2
ABI 3500xl	38	2		1	3	2		2		8			8	1		4		4	1			25	4
Other	1																						

#### 2.4.4 Short Tandem Repeat (STR) Analysis Software

For genotyping software (PP#19–20), 83 labs use Applied Biosystems GeneMapper ID-X (particularly versions 1.4, 1.5, and 1.6). For smoothing method (PP#21), 72 labs use Gene-Mapper ID-X light smoothing; 11 labs do not typically apply smoothing. The Local Southern method for allele-size calling (PP#22) is used by 76 labs; other methods used are 3rd order least square (*n* = 4) and Global Southern (*n* = 2).

#### 2.4.5 Profile Analysis

Table 7 describes participating laboratories’ policies regarding default analytical threshold (AT) and stochastic threshold (ST) values, and the situations in which they are permitted to vary said values. Default AT values reported by labs (PP#24) range from 40 to 200 relative fluorescence units (RFUs) (mean 96 RFUs, std dev 39 RFUs, median 100 RFUs). Default ST values reported by labs (PP#27) range from 150 to 1250 RFUs (mean 456 RFUs, std dev 238 RFUs, median: 400 RFUs). Note that AT and ST values are a function of amp kit, number of cycles, and other factors, so caution should be used in comparing AT and ST across different Amp/CE settings. Note also that because the CE instrument was a “check all that apply” question asked in RC, and AT and ST questions were free text entries asked in P&P, we do not have the data to reliably report the intersection between the responses.

**Table 7:** Laboratory usage of analytical (AT) and stochastic (ST) thresholds during DNA mixture profile analysis. “Check all that apply” responses may total greater than 86. See the P&P-CSQ dataset [1] for details about combinations of responses that were selected.

			# Labs	
Category			AT	ST
Established threshold value (PP#23, PP#26)	Single default value	Never varies	52	51
		Can be changed at analyst's discretion	3	0
		Can be changed with technical leader approval	2	0
	No single default value		28	10
	Do not use		0	24
	N/A: inconsistent		**1**	**1**

Situations permitting threshold to vary (check all that apply) (PP#25, PP#28) *Limited to labs that have no single default value*	By dye channel		27	4
	By peak height of profile		2	0
	By injection time/voltage		1	4
	By locus		0	2
	By AT used		0	1
	Other explanation		5	4

Table 8 details participating laboratories’ SOPs for handling stutter and other artifacts. Over three-quarters of labs use stutter filters at some point during their analyses of DNA mixtures (either always on or both on and then off). All laboratories but one (*n* = 85) have policies for handling artifacts other than stutter, such as pull-ups, spikes, or dye blobs (PP#30). Nearly all labs handle artifacts via manual evaluation and labeling by the analyst (sometimes paired with re-analysis and/or automated evaluation of artifacts). All but nine labs consider two or more artifacts in their interpretations, most commonly high stutter and/or tri-alleles; about one-quarter of the participating labs consider five or more types of artifacts.

**Table 8:** Laboratory procedures for handling stutter and other artifacts. “Check all that apply” responses may total greater than 86. See the P&P-CSQ dataset [1] for details about combinations of responses that were selected.

Category		# Labs
Use of stutter filters (PP#29)	On	43
	Once with filters on and once with filters off	29
	Off	11
	N/A: inconsistent	**3**

Procedures for handling other artifacts (check all that apply) (PP#31) *Limited to the 85 labs that have procedures for handling other artifacts*	Manual evaluation and labeling by analyst	84
	*Re*-analysis, if possible	25
	Automated evaluation and labeling by software	19
	Other	2

Artifacts/characteristics that may lead to differences in interpretation (check all that apply) (PP#32) *Limited to the 85 labs that have procedures for handling other artifacts*	High stutter	68
	Tri-alleles	67
	Non-specific amplification peak	36
	Pull up	32
	Non-human peaks	28
	Spike	22
	Dye blobs	15
	Pull down (raised baseline)	13
	Other	9

#### 2.4.6 Methods of Assessing Number of Contributors (NoC)

As shown in Table 9, nearly all laboratories that participated in this study manually assess the number of contributors (NoC) in a DNA mixture, rather than using software or another analysis tool. When evaluating number of contributors in a mixture, participating laboratories cited 9 of the listed indicators, on average (minimum: 1, maximum: 13, median: 10).

**Table 9:** Method for assessing number of contributors in a DNA mixture (*n* = 86 labs) and indicators considered during manual determination NoC. “Check all that apply” responses may total greater than 86. See the P&P-CSQ dataset [1] for details about combinations of responses that were selected.

Category		# Labs
Method for assessing NoC (PP#38)	Assess number of contributors manually	83
	Other	3

Indicators taken into consideration during manual determination of the NoC (select all that apply) (PP#39) *Limited to the 83 labs that manually assess NoC*	Maximum Allele Count (MAC) per locus	81
	Relative peak heights (peak height ratios and possible shared/stacked alleles)	80
	Peak heights (RFU)	79
	Sex determining markers	75
	Expected stutter ratios	69
	Information below the analytical threshold	69
	Presence of degradation	61
	Overall level of data (peak heights in relation to laboratory validated thresholds)	57
	Peak morphology (e.g., CE resolution; unresolved microvariants; peak shouldering)	54
	Presence of inhibition	50
	Discriminating potential/variability of loci (or allele frequency)	39
	Quantitation data	35
	Total allele count in profile	29
	Other	2

Table 10 outlines various policies and procedures related to evaluating NoC. Just over two-thirds of participating laboratories are permitted to evaluate a mixture under different assumed/estimated numbers of contributors and just over half are permitted to change assumed NoC after conducting statistical analysis. There is notable variation in how laboratories handle reporting when there is uncertainty in the assumed number of contributors to a DNA mixture.

**Table 10:** Policies and procedures related to evaluating NoC and documenting and/or updating these assessments.

Category	Response	# Labs
Permitted the option to evaluate a mixture under different assumed numbers of contributors (PP#33)	Yes	60
	No	24
	N/A: inconsistent	2

Required to determine and record NoC BEFORE comparison to the victim, consensual partner, and/or expected contributor (PP#34)	Yes, but are then permitted to change the assumed NoC after such comparison	43
	Yes, and the assumed NoC cannot be changed after such comparison	8
	No	31
	N/A: inconsistent	4
Required to determine and record NoC BEFORE comparison to the POI (PP#35)	Yes, but are then permitted to change the assumed NoC after such comparison	38
	Yes, and the assumed NoC cannot be changed after such comparison	36
	No	7
	N/A: inconsistent	5

Permitted to change the assumed NoC AFTER conducting statistical analyses (PP#36)	Yes	46
	No	38
	N/A: inconsistent	2

How to report if there is uncertainty in the assumed number of contributors in a mixture (PP#37)	The mixture is not suitable for comparison due to the unknown NoC	24
	Report only with respect to major contributors	15
	Report the statistical value that provides the lowest evidential weight	9
	Report one statistical value that accounts for variable number of contributors (VarNoC)	4
	Report multiple statistical values	3
	Other	24
	N/A: inconsistent	7

#### 2.4.7 Differentiation of Major Versus Minor Contributors

According to Table 11, two-thirds of participating laboratories differentiate between major and minor contributors for DNA mixtures; however, the specific methods of doing so and/or thresholds used vary between laboratories (both for mixtures interpreted to include two contributors and those interpreted to include more than two contributors).

**Table 11:** Procedures for differentiating between major and minor contributors in DNA mixtures.

Category		# Labs
Differentiate between major and minor contributors (PP#40)	Yes	56
	No	29
	N/A: inconsistent	1

Methods for differentiating between major and minor contributors for mixtures interpreted to include two contributors (PP#41) *Limited to 56 labs that differentiate majors from minors*	4:1 ratio	12
	3:1 ratio	11
	Less than 3:1 ratio	6
	Distinguished visually based on peak heights without calculation (tallest peaks)	7
	Other	16
	N/A: inconsistent	4

Methods for differentiating between major and minor contributors for mixtures interpreted to include more than two contributors (PP#42) *Limited to 56 labs that differentiate majors from minors*	Yes, manually (e.g. distinguished visually based on peak heights)	36
	Yes, using software	12
	No	8

#### 2.4.8 Suitability for Interpretation

The suitability of a DNA mixture for interpretation and/or comparison is dependent upon many factors, including most primarily the total DNA quantity and the number of contributors. Note that Section 4.4.1 outlined participating laboratories’ policies and procedures with respect to suitability based upon quantification information.

Table 12 shows that nearly 90% of labs limit their interpretation/comparison of DNA mixtures based upon a maximum total number of contributors (most commonly 4); however, fewer labs limit based upon a maximum number of unknown contributors or minor contributors.

**Table 12:** Assessments of suitability with respect to number of contributors.

Category			# Labs
Limit interpretation and/or comparison based on a maximum total NoC (PP#43)	Yes	4 total contributors	50
		5 total contributors	12
		3 total contributors	12
		6 total contributors	1
		2 total contributors	1
	No		8
	N/A: inconsistent		2

Limit interpretation and/or comparison based on a maximum number of unknown contributors (PP#44)	Yes	4 unknown contributors	13
		3 unknown contributors	6
		2 unknown contributors	2
	No		61
	N/A: inconsistent		4

Limit interpretation and/or comparison based on a maximum number of minor contributors (PP#45)	Yes	2 minor contributors	4
		1 minor contributors	3
		4 minor contributors	2
		3 minor contributors	1
	No		45
	N/A: inconsistent		2

Of the 56 labs that differentiate between major and minor contributors (in PP#40), 31 permit interpretation of the major contributor if a mixture has ONE major contributor and an unknown number of minor contributors (PP#46); 34 permit interpretation of the major contributor if a mixture has TWO OR MORE major contributors and an unknown number of minor contributors (PP#47).

Approximately half of the labs (*n* = 40) require a minimum number of loci with data in order to interpret a DNA mixture (PP#48), ranging from 2 to 15 loci with data; the most frequent minimum values were 6 or 8 loci with data. Few labs (*n* = 6) require a minimum number of alleles called with data in order to interpret a mixed DNA profile (PP#49), ranging from 2 to 16 alleles called.

Just 13 participating laboratories permit DNA mixtures to be considered suitable for exclusion, but not inclusion/statistical analysis (PP#50).

#### 2.4.9 Types of Categorical Conclusions Reported

Table 13 describes participating laboratories’ usage of various categorical conclusions for reporting the results of their comparisons between reference profiles and DNA mixtures. In general, nearly all labs report exclusion and inclusion (or equivalent—see PP#53–55 for details about other equivalent terms), but approximately one-quarter of labs do not report inconclusive as a conclusion.

**Table 13:** Usage of categorical conclusions for reporting the results of DNA mixture comparisons.

Category	“Exclusion” or “Excluded” (PP#53)	“Inclusion” or “Included” (PP#54)	“Inconclusive” (PP#55)
Report the term as a conclusion	69	37	37
Use a different, but equivalent term	9	24	22
Use multiple conclusions that collectively correspond	5	19	0
Do not report the term or equivalent term as a conclusion	2	3	21
N/A: inconsistent	1	3	6

Table 14 details the number of laboratories that permit exclusion and/or inclusion of a person of interest as a potential contributor to a mixture based SOLELY upon manual review of the electropherograms and WITHOUT additional statistical support. The majority of labs permit manual exclusion by analyst discretion or under specific circumstances. Conversely, nearly three-quarters of labs do NOT permit manual inclusion of a POI without additional statistical support under any circumstances. (Note: with respect to manual inclusions, the SWGDAM Interpretation Guidelines [6] require that “except for a reasonably assumed contributor, the laboratory shall perform statistical analysis in support of any inclusion.”).

**Table 14:** Laboratory policies regarding manual exclusion and/or inclusion of a POI as a potential contributor to a mixture based SOLELY upon manual review of the electropherograms.

Category			# Labs
Manual exclusion policies (PP#56) *Limited to the 84 labs that report “exclusion” or equivalent*	Permitted	Manual exclusion is permitted by analyst discretion	40
		Manual exclusion is permitted in some specified circumstances if some alleles are below ST	8
		Manual exclusion is permitted only if ALL alleles are above ST	3

	Manual exclusion is not permitted		13
	Other manual exclusion policy		15
N/A: inconsistent		5

Manual inclusion policies (PP#57) *Limited to the 83 labs that report “inclusion” or equivalent*	Permitted	Manual inclusions can be reported for major or minor contributors	15
		Manual inclusions can only be reported for a (single) major contributor	2

	Manual inclusion is not permitted		62
N/A: inconsistent		4

#### 2.4.10 Conditioning

As detailed in Table 15, nearly all participating laboratories’ SOPs include the option to use conditioning (assuming the presence of a victim, consensual partner, and/or expected contributor) during interpretation, comparison, or statistical analysis, often under certain criteria. Before conditioning, the majority of labs require statistical analysis to be run with respect to the POI and/or require the analyst to evaluate the victim/consensual partner/expected contributor profile to determine whether there is support for inclusion.

**Table 15:** Use of conditioning during DNA mixture interpretation, comparison, and/or statistical analysis.

Category			# Labs
Allow the option to use conditioning (PP#60)	Yes		84
	No		2
Prior to conditioning, require statistical analysis to be conducted with respect to the POI (PP#61) *Limited to the 84 labs that allow conditioning*	Yes		10
	No		72
	N/A: inconsistent		2

Prior to conditioning, require manual interpretation and/or statistical analysis to support the inclusion of the victim, consensual partner, and/or expected contributor (PP#62) *Limited to the 84 labs that allow conditioning*	Yes	Only manual interpretation (non-statistical) is required	49
		Both manual interpretation and statistical analysis are required	9
		Only statistical analysis is required	4

	No (neither manual interpretation nor statistical analysis)		14
N/A: inconsistent		8

Report statistics both before and after conditioning (PP#63) *Limited to the 84 labs that allow conditioning*	Never: we condition but only report after		56
	Rarely		20
	Often		4
	N/A: inconsistent		4

#### 2.4.11 Statistical Approach

Table 16 details laboratory policies for reporting statistical analyses of DNA mixtures. Most labs (*n* = 71) report some type of LR (continuous, binary, and/or semi-continuous), 27 of which use LRs in addition to another type of statistic. Of the 13 labs that report CPI/CPE or RMP/mRMP but do not report LRs, 4 use RMP/mRMP only, and 1 uses CPI/CPE only; 12 of these 13 labs are in the U.S. The 2 labs that indicated that they do not report any statistics are not in the U.S. Approximately half of the participating labs have the option of computing more than one type of statistical value, but the criteria for determining which value to report for a given case varies notably by laboratory.

**Table 16:** Policies for reporting the results of statistical analyses of DNA mixtures (*n* = 86 labs). “Check all that apply” responses may total greater than 86. See the P&P-CSQ dataset [1] for details about combinations of responses.

Category		# Labs
Types of statistical values reported to support interpretations and comparisons of DNA mixtures (select all that apply) (PP#51)	Continuous Likelihood ratios (LRs)	58
	Random match probability (RMP)	34
	Combined probability of inclusion/exclusion (CPI/CPE)	21
	Binary LRs	17
	Modified RMP (mRMP)	17
	Semi-continuous LRs	5
	Other	5
	We do not report any statistics	2

Criteria for determining which statistical value to report when more than one reported (select all that apply) (PP#52)	N/A: only use one statistical method	43
	Specified in SOPs based on mixture attributes or sample types	23
	Use probabilistic genotyping when possible, but cannot for some cold cases due to data limitations	11
	Analyst discretion based on mixture attributes	11
	Use probabilistic genotyping for complex mixture, but RMP/mPMP for simple mixtures	7
	Technical leader discretion based on mixture attributes	2
	Use probabilistic genotyping for complex mixtures, but CPI/CPE for simple mixtures	1
	Specified by requester	1
	Other	8
	N/A (do not report any statistics)	2

#### 2.4.12 Population Databases

Most labs (52) routinely use the NIST population databases, 25 labs use the FBI population databases (one of which also uses NIST), and ten labs use internal or other databases (one of which also uses FBI) (PP#64). Over three-quarters of labs routinely compute statistics for at least three populations (PP#65): 77 labs use Caucasian, 71 use African American, and 63 use Hispanic population databases routinely; 24 labs use a Combined Asian database in their computations. When multiple databases are used for computing statistics (PP#67), nearly two-thirds of labs (*n* = 57) report the statistical value that provides the lowest evidential weight (i.e., most conservative result); 19 labs report all computed statistics. Approximately one-third of laboratories are permitted flexibility in using additional non-standard population databases based upon case relevant circumstances (PP#66). In the computation of statistics, over three-quarters of laboratories (*n* = 68) are not permitted to change theta from the default value specified in their SOPs, irrespective of the case scenario (PP#68).

#### 2.4.13 Use of Probabilistic Genotyping

At the time of this study, approximately two-thirds of participating laboratories were using PGS for operational casework, and another 16 labs were in the process of validating PGS for use (Table 17). STRmix was used by the vast majority of labs currently using PGS. Thirteen versions (2.3 −2.9) of STRmix were reported by the participants, with versions 2.5.11 and 2.7 most commonly used (PP#71).

**Table 17:** Laboratory use of probabilistic genotyping software for DNA mixture casework.

Question	Response	# Labs
Use of PGS (PP#69)	Yes, currently in use for casework	58
	Yes, we plan to implement in 0–2 years and we are currently in the process of validation	16
	Yes, we plan to implement in 0–2 years and we have not yet started validation	6
	Yes, we plan to implement in 3 or more years	3
	No	3

PGS (PP#70) *Limited to the 58 labs that currently use PGS*	STRmix	52
	TrueAllele	2
	EuroForMix	1
	LRmix / LRmix Studio	1
	Other	2

Of the 58 labs currently using PGS, 42 report the highest posterior density (HPD), a statistic adjusted to account for the variation introduced due to the Monte Carlo sampling in the software (Table 18). When statistics are computed in a case to account for multiple propositions, laboratories vary notably in their policies regarding which statistic(s) to report—in general, the decision is based upon the case scenario, as outlined in Table 18.

**Table 18:** Detailed LR statistics that laboratories report and methods for reporting statistics given multiple propositions. “Check all that apply” responses may total greater than 86. See the P&P-CSQ dataset [1] for details about combinations of responses that were selected.

Category		# Labs
Specific LR statistics reported (check all that apply) (PP#72) *Limited to 58 labs that currently use PGS*	Highest posterior density (HPD)	42
	Sub-source level (weights the probabilities for all contributors to the mixture)	13
	Unified (combined related and unrelated contributors)	9
	Stratified (combined population groups)	6
	LRs that consider different NoC in the propositions (e.g., VarNoC)	5
	Sub-sub-source levels (a particular contributor within a set of contributors to the mixture)	4
	Point estimate	3
	None of the above	2
	Average of replicates	1

Methods for reporting statistics given multiple propositions (PP#74) *Limited to 58 labs that currently use PGS*	Depends on case scenario	32
	Report all values	12
	Report the most conservative result (lowest LR) for each individual POI	7
	Other	3
	N/A: inconsistent	4

Tables 19 and 20 detail participating laboratories’ SOPs regarding their PGS stutter modeling and other parameters. The vast majority of labs that currently use PGS use a system that models stutter—most commonly forward and back stutter of 1 repeat unit. PGS labs often permit software parameters to vary (most commonly the number of Markov chain Monte Carlo (MCMC) repetitions/iterations), a decision that is commonly based upon software diagnostics or when the first iteration gave unintuitive results. However, analysts generally cannot re-run profile deconvolution and/or statistical analysis based upon the LR value (52/58 labs; PP#75).

**Table 19:** PGS stutter modeling (*n* = 86 labs). “Check all that apply” responses may total greater than 86. See the P&P-CSQ dataset [1] for details about combinations of responses that were selected.

Category		# Labs
Use of stutter modeling by PGS (PP#76) *Limited to 58 labs that currently use PGS*	Yes	54
	No	4

Types of stutter modeled by PGS (check all that apply) (PP#77) *Limited to 58 labs that currently use PGS*	Backstutter (−1 repeat unit)	53
	Forward stutter (+1 repeat unit)	51
	Backstutter (−2 bases, or other partial repeat)	19
	Backstutter (−2 repeat units)	16
	Forward stutter (+2 bases, or other partial repeat)	4
	Forward stutter (+2 repeat units)	2

**Table 20:** Policies regarding the PGS parameters that are permitted to vary and the circumstances under which these changes are permitted. “Check all that apply” responses may total greater than 86. See the P&P-CSQ dataset [1] for details about combinations of responses that were selected.

Category		# Labs
Parameters permitted to vary depending upon sample or case (check all that apply) (PP#73) *Limited to 58 labs that currently use PGS*	MCMC repetitions	33
	Range of contributor mixture proportions observed in the DNA profile	20
	Maximum level of degradation	17
	Stutter variance	7
	Peak variance	6
	Other	3
	None of the above	13

Software parameters that can be changed based on profile characteristics (check all that apply) (PP#78) *Limited to 58 labs that currently use PGS*	MCMC iterations	36
	Burn-in accepts per chain	29
	None of the above	14
	MCMC chains	2
	Random walk distance	2

Reasons to make software parameter changes (check all that apply) (PP#79) *Limited to 58 labs that currently use PGS*	Software diagnostics	40
	First run through software gave unintuitive results	37
	Mixture complexity (e.g., number of contributors)	16
	Mixture quality (e.g., artifacts, degradation, inhibition, etc.)	10
	Mixture quantity (e.g., low quantity, low peak heights, etc.)	8

#### 2.4.14 Use of Non-Probabilistic Genotyping Software

Among the 28 participating labs that were not using PGS at the time of the study, Popstats is used by 13 labs and 6 labs use an internally developed system for analyzing DNA mixtures (PP#80). The majority of laboratories that compute CPI/CPE and/or RMP/mRMP statistics use the methods detailed in the literature [7,8] as “best practices” (PP#81-82).

Table 21 details various policies and considerations for computing statistical values for DNA mixtures using non-PGS. The majority of participating labs have the option of computing restricted or unrestricted statistics, depending upon the sample or profile. Most Binary LR users do not use loci that include potential dropout in their computations. There is notable variation in how labs handle loci that have indistinguishable peaks, with approximately half dropping the locus all together and the remainder using the locus (either in full or part).

**Table 21:** Policies and Procedures regarding statistical computations for non-PGS users.

Question	Response	# Labs
When calculating statistics, do you calculate restricted or unrestricted statistics? (For Binary LR, CPI/CPE, or RMP/mRMP users) (PP#83)	Use restricted if can pull one or more majors, otherwise unrestricted	14
	Depends on the profile	13
	Always unrestricted	8
	Always restricted	6
	Use restricted if conditioned on an expected contributor, otherwise unrestricted	4
	N/A (lab does not report Binary LR, CPI/CPE, RMP/mRMP)	39
	N/A: inconsistent	2

Do you utilize loci that include potential dropout? (For Binary LR users) (PP#84)	No (drop the locus)	11
	Yes (using a 2P calculation)	4
	Other	2
	N/A (lab does not report Binary LR)	68
	N/A: inconsistent	1

Do you utilize loci that include peaks that cannot be distinguished either as alleles or stutter? (For any non-PGS users) (PP#85)	No (drop locus)	15
	Yes (use locus and include such peaks in the calculations)	10
	Yes (use locus but drop such peaks from the calculations)	3
	N/A (lab uses PGS)	58

#### 2.4.15 Reporting Language

As shown in Table 22, participating laboratories vary notably in whether they use LR thresholds to differentiate between inconclusive/inclusion and/or inconclusive/exclusion, and when they do, the thresholds also exhibit large variability. Of the 70 labs that report LRs, 31 do not use a verbal equivalent in reporting; 38 do use a verbal equivalent, of which 29 use the SWGDAM verbal equivalent scale (PP#92–93).

**Table 22:** Use of LR thresholds to differentiate categorical conclusions.

Question	Response	Free Text Entry	# Labs
Do your SOPs define a specific LR value used as a threshold to differentiate conclusions of inconclusive and included? (PP#90)	Yes, we define a single LR threshold	2	6
		1000	8
		Other values	8

	No we report conclusions of “included” but our SOPs do not define a specific LR threshold		13
	Yes, but there are multiple LR thresholds		8

	No we do not report conclusions of “Included”		6
N/A (do not report “inclusion”, “inconclusive”, and/or LR statistics)		32
N/A: inconsistent		5

Do your SOPs define a specific LR value used as a threshold to differentiate conclusions of inconclusive and excluded? (PP#91)	Yes, we define a single LR threshold	0.001	5
		0.01	8
		Other values	12

	No we report conclusions of “excluded” but our SOPs do not define a specific LR threshold		13
	Yes, but there are multiple LR thresholds		7

	No we do not report conclusions of “excluded”		3
N/A (do not report “exclusion”, “inconclusive”, and/or LR statistics)		31
N/A: inconsistent		7

#### 2.4.16 Interpretation and Validation Guidelines

Table 23 shows which DNA mixture-related standards and guidelines that participating labs follow. The vast majority of participating labs comply with the SWGDAM Interpretation Guidelines.

**Table 23:** Compliance with DNA mixture-related standards and guidelines.

Category	Yes	No	Don't know	N/A (non-PGS lab)	N/A: inconsistent
SWGDAM Interpretation Guidelines for Autosomal STR Typing by Forensic DNA Testing Laboratories (PP#86)	80	0	5		1

ASB Standard for Forensic DNA Interpretation and Comparison Protocols (ANSI/ASB Standard 040, 2019) (PP#87)	50	2	28		6

ASB Standard for Validation Studies of DNA Mixtures, and Development and Verification of a Laboratory's Mixture Interpretation Protocol (ANSI/ASB Standard 020, 2018) (PP#88)	45	6	28		7

ASB Standard for Validation of Probabilistic Genotyping Systems (ANSI/ASB Standard 018, 2020) (PP#89)	36	6	25	12	7

### 2.5 Casework Scenario Questionnaire (CSQ) Responses

This section provides a summary and overview of the results from *Phase 2: Casework Scenario Questionnaire* that are included in detail in the P&P-CSQ dataset [1]. Each section below indicates the specific question numbers being presented, prefixed with “CS#” (*CS Questionnaire*). As discussed in Section 4.2 (Test Yield), all results in this section are based on the responses from the 83 forensic laboratories that completed the CSQ. Responses are reported by laboratory, using the majority response for laboratories with multiple subunits.

#### 2.5.1 Case Information

Table 24 details the types of information that participating labs usually have available during interpretation of DNA mixtures, as well as the mechanisms (if any) for filtering the information that is available to the analyst. The majority of labs have the results of the sexual assault medical exam report available, whereas approximately one-quarter to one-third of labs have access to investigator statements, law enforcement case files, and/or the results of other forensic analyses. Interestingly, approximately 10% of labs indicated that they do not have any of the listed sources of information available to them during their interpretations. Over 80% of participating labs do not have a mechanism for filtering or triaging case information (e.g., sequential unmasking, blinding, etc.) so that all available information is provided to the analyst up front.

**Table 24:** Types of information and mechanisms of filtering information available to DNA analyst. “Check all that apply” responses may total greater than 83. See the P&P-CSQ dataset [1] for details about combinations of responses that were selected.

Question	Response	# Labs
When the following types of information exist in a case, what do you USUALLY have available during interpretation of DNA data? (check all that apply) (CS#02)	Sexual assault medical exam report	60
	Investigator statements	39
	Case file (from law enforcement)	25
	Results of other forensic analyses	20
	Crime scene notes	16
	Complainant or victim statements	9
	Images of the crime scene	7
	Witness statements	5
	CCTV footage	1
	None of the above	8

Does your laboratory have a mechanism for filtering or selecting information available to the DNA analysts? (CS#03)	No: all case information is provided to the DNA analyst up front	68
	Yes: a case manager or other individual triages or restricts case information	9
	Yes: a LIMS or other digital system/process triages or restricts case information	3
	N/A: inconsistent	3

In general, the 69 participating labs that report LRs do not often get information directly from the prosecution or the defense to help them formulate their propositions (CS#04–05): 46 labs never get such information from the prosecution, and 19 occasionally do; 57 labs never get such information from the prosecution, and 11 occasionally do.

#### 2.5.2 Sample Sources/Origins

In terms of the various types of DNA mixture samples encountered in casework (CS#07), the vast majority of labs often receive sexual assault kits (SAKs) (76 often, 6 occasionally) and touch DNA casework (77 often, 6 occasionally), and about half of labs often encounter casework involving other bodily fluids (40 often, 34 occasionally).

Laboratories generally know the source of a DNA mixture, the crime type, and/or the origin of DNA samples (for SAKs and touch samples). The vast majority of participating labs know (at least often) whether a DNA mixture is from a SAK, touch sample, or body fluid sample (CS#06)—nearly two-thirds of labs indicated that they always know this information. Similarly, labs nearly always know the crime type (CS#10) for non-SAK samples, and the origin of SAK samples (CS#08); all labs indicated that they know (at least often) the origin of touch DNA samples (CS#09).

#### 2.5.3 Reference Samples

With respect to reference samples, reference samples from persons of interest (POIs) are differentiated from assumed contributors (e.g., victim, consensual partner, expected contributor) for 78 labs; this is sometimes true for the other 5 labs (CS#11). For sexual assault kits, analysts are always informed which reference samples are from victims vs consensual partners in every participating lab (CS#12).

#### 2.5.4 Analysis

Table 25 shows that nearly all labs use conditioning when analyzing SAK samples; additionally, the majority of labs condition for touch samples that originate from clothing with a primary wearer and handled items with a primary user.

**Table 25:** Types of samples for which conditioning is usually used during analysis. “Check all that apply” responses may total greater than 83. See the P&P-CSQ dataset [1] for details about combinations of responses that were selected.

Question	Response	# Labs
Types of samples that USUALLY use conditioning (check all that apply) (CS#13)	SAK samples	81
	Clothing with primary wearer	67
	Handled items with primary user	55
	Bodily fluid mixtures	24
	Briefly touched items	2
	None of the above	1

Profile-specific LR distributions (such as STRmix DBLR Explore Deconvolution [9]) are never used by 61 of the 68 labs that compute LRs (CS#14). Mixture to mixture comparisons are never conducted by 55 labs; 22 labs occasionally do mixture to mixture comparisons, and 5 often do (CS#15).

#### 2.5.5 Sexual Assault Kit Samples

As shown in Table 26, participating labs at least occasionally encounter sexual assault samples that include 3 or more unknown contributors, in addition to the victims and/or a consensual partner.

**Table 26:** Distribution of DNA mixture casework in which the mixture and reference profile(s) suggest the presence of various numbers of contributors, where *V*=victim, C=consensual partner, *U*=unknown contributors. For example, “1 V, ≥ 1C, ≥ 3U” asks how often casework is encountered with 1 victim, at least 1 consensual partner, and at least 3 unknowns.

	SAK contributors					
Category	1 V***, 0C,*** 1 U *(CS#16A)*	1 V***, 0C, ≥*** 2 U *(CS#16B)*	1 V***, 0C, ≥*** 3 U *(CS#16C)*	1 V***, ≥ C1,*** 1 U *(CS#16D)*	1 V***, ≥ 1C, ≥*** 2 U *(CS#16E)*	1 V***, ≥ 1C, ≥*** 3 U *(CS#16F)*
Often	74	28	4	31	5	1
Occasionally	5	49	62	42	64	48
Never	0	0	13	1	7	31
N/A: lab does not encounter SAKs	1	1	1	1	1	1
N/A: inconsistent	3	5	3	8	6	2

Most labs (*n* = 70) indicated that in sexual assault kit samples other than vaginal swabs received in casework, the victim profile is often present; 8 labs indicated the victim profile is always present (CS#17). In all sexual assault kit samples received in casework, most labs (*n* = 78) indicated the sperm fraction DNA profile is occasionally degraded (CS#18).

#### 2.5.6 Touch DNA Samples

Of their DNA mixture casework that comes from touch DNA samples, the majority of participating labs indicate that they at least occasionally encounter samples with up to 5 or more unknown contributors (Table 27). Labs occasionally or often receive degraded touch DNA samples, and touch DNA samples are occasionally or often submitted with reference samples.

**Table 27:** Distribution of touch DNA casework with respect to the number of unknown contributors (estimates based on mixture and reference profiles), as well as how often touch DNA samples are degraded, and how often touch DNA samples are provided with reference samples.

	Number of unknown contributors				Touch DNA sample details	
Category	***2*** *(CS#19A)*	***≥ 3*** *(CS#19B)*	***≥ 4*** *(CS#19C)*	***≥ 5*** *(CS#19D)*	***Degraded*** *(CS#20)*	***Reference*** *(CS#21)*
Always					2	4
Often	65	69	41	10	41	34
Occasionally	12	9	32	61	37	40
Never	0	2	4	7	1	0
Invalid response (software artifact)		1				
N/A: inconsistent	6	2	6	5	2	5

#### 2.5.7 Contributor Ratios and Amount of DNA

Table 28 reports the frequency with which participating laboratories encounter various contributor ratios in casework—all but one lab encounter a range of ratios up to 10:1 or greater at least occasionally. With respect to DNA quantity, most labs will interpret DNA mixture samples with 10–50 pg of DNA, but just over half of the participating labs will not interpret samples with less than 10 pg of DNA.

**Table 28:** Distribution of DNA mixture casework by contributor ratios, and by quantity of DNA.

	Ratio between the highest and second highest contributor				How often do you INTERPRET samples with total DNA quantity:	
Category	***1:1*** *(CS#22A)*	***2:1–4:1*** *(CS#22B)*	***5:1–10:1*** *(CS#22C)*	***>10:1*** *(CS#22D)*	***10–50 pg (0.01–0.05*** ***ng)*** *(CS#23)*	***< 10 pg (0.01*** ***ng)*** *(CS#24)*
Often	34	67	53	34	24	5
Occasionally	43	13	26	44	38	33
Never	1	0	1	1	15	42
N/A: inconsistent	5	3	3	4	6	3

#### 2.5.8 Related Contributors

Several CSQ questions address how often participating laboratories encounter DNA mixture casework that involves first degree biological relatives (i.e., parent, sibling, or child). Nearly all labs occasionally encounter casework in which multiple POIs are (or are alleged to be) related (CS#25), as well as casework in which the victim and a POI are (or are alleged to be) related (CS#26). About three-quarters of labs occasionally encounter scenarios in which a POI in a crime alleges that a first degree relative was the actual POI instead (aka “my brother did it”, CS#27)—the remainder of the participating labs indicated that they never encounter this scenario in their DNA mixture casework.

Tables 29 and 30 detail how participating labs conduct their statistical analyses of DNA mixtures when the DNA profile or case scenario suggests related contributors. Approximately half of participating labs do nothing different when the DNA profile or case scenario suggest that the victim and POI are related. Similarly, just under half of participating labs do nothing different from usual when a POI alleges that a first degree relative is an alternate POI.

**Table 29:** Actions taken by laboratories when the DNA profile or Case Scenario suggest that the victim and the POI are 1st degree biological relatives.

Category	DNA Profile (CS#28)	Case Scenario (CS#29)
We do nothing different (than if they were not related)	42	41
We use multiple propositions or adjust the propositions used in statistical analysis	8	12
We address this by using unified LRs	7	8
Other	19	14
N/A: inconsistent	7	8

**Table 30:** Actions taken by laboratories when the POI alleges that a 1st degree biological relative was an alternate contributor. (Question CS#30).

Category	Alternate Contributor
We do nothing different from usual in this situation	35
We do not report stats until references from the related individuals are submitted	5
We address this by using unified LRs	4
We report both types of stats (i.e., non-related and related both)	3
Other	12
N/A: lab never encounters casework in which POI alleges 1st degree relative as alternate POI	16
N/A: inconsistent	8

## 3 Experimental Design, Materials and Methods

It is generally recognized that there is variation in the interpretation and statistical reporting of “complex” DNA mixtures that include three or more contributors [5,10]. A variety of factors—including degradation or inhibition, low DNA quantity, disproportionate contributor ratios, allele sharing, and number of contributors—can impact the complexity of these DNA mixture interpretations and comparisons which may, in turn, influence the reliability of the reported conclusions and statistical analyses. In fact, numerous DNA mixture studies conducted over the past several decades have demonstrated that laboratories vary in their interpretations and reporting for DNA mixture samples (see [5] for a detailed review). However, nearly all of these studies were conducted using traditional binary approaches (e.g., random match probabilities (RMP) and/or combined probabilities of inclusion/exclusion (CPI/CPE)) prior to the advent of probabilistic genotyping (PG) software, which significantly changes the interpretation of DNA mixtures [11,12]. The President's Council of Advisors on Science and Technology (PCAST) asserted that “substantially more evidence is needed to establish foundational validity” of DNA mixture interpretation across broader settings and using PG software [10,13]. The purpose of DNAmix 2021 was to conduct a rigorous, large-scale independent study to evaluate the extent of consistency and variation among forensic laboratories in interpretations and statistical analyses of DNA mixtures, and to assess the effects of various potential sources of variability. In other words, if two laboratories are given the same mixture (as an electropherogram) and the same person of interest, how consistent are the statistical responses and categorical interpretations? And, what factors explain any differences in responses?

To this end, this study utilized a multi-phased approach (see Section 5.1) designed to assess multiple potential sources of variability in the interpretations and statistical analyses of DNA mixtures. Participation in DNAmix 2021 was open to all forensic laboratories that conduct DNA mixture interpretation as part of their SOPs (see Section 5.2); non-U.S. laboratories were welcome to participate if they report their interpretations in English.

This paper details the data collected in the first two phases: the *Policies and Procedures Questionnaire* and the *Casework Scenarios Questionnaire*. These questionnaires (see Section 5.3) provide dual benefit to this study by describing the state of the field in terms of laboratories’ policies, procedures, and case-specific decisions in the assessment of DNA mixtures, as well as the nature of their DNA mixture casework. In addition, the questionnaires illuminate the large set of factors that may introduce variability in the assessment of DNA mixtures (from suitability determination to number of contributors assessment, through comparison and statistical analysis).

All study materials (e.g., questionnaire/subtest questions and response options, instructions, glossary, etc.) were created in collaboration with a variety of forensic DNA analysts, academics, and researchers via two consultation groups (see Section 5.4): the DNAmix Working Group and DNAmix Advisory Group. The main purpose of these consultation groups was to ensure that materials provided to participants were logical, understandable, reasonably comprehensive, and broadly applicable/appropriate across kits, software, and statistics.

### 3.1 Study Design

The study was composed of four phases (Note that here we only report on the results of Phases 1 and 2):

(1) *Policies and Procedures (P&P) Questionnaire* — Online questionnaire to assess laboratory policies and procedures relevant to DNA mixture interpretation (notably systems, types of statistics reported, and parameter settings used).
(2) *Casework Scenario (CS) Questionnaire* — Online questionnaire to assess analysis procedures or decisions that may vary depending upon the case scenario, and to assess the nature of mixture casework.
(3) *Number of Contributors (NoC) Subtest* — Assessment of suitability and number of contributors, given electropherogram data. A total of 21 mixtures were used in this subtest, out of which each participant was assigned 12 mixtures.
(4) *Interpretation, Comparison, and Statistical Analysis (ICSA) Subtest* — Interpretations and statistical analyses, given electropherogram data for 8 mixtures, each provided with DNA profiles of potential contributors. All participants received the same 8 mixtures.

In addition to the four main phases of the study, a series of short supplemental questionnaires were also distributed to participants to aid in the selection of “Amp/CE settings”^4^ used in creating mixtures, allow laboratories to select which versions of electropherograms they wished to use for their assessments, indicate how the selected settings compared to their SOPs, and report which specific population databases/settings they used for their statistical analyses (if applicable).

To conduct this study, a custom DNAmix 2021 website was created in order to provide participants with access to all online questionnaires and subtests, allow for download of electropherogram data, and provide a user interface for reporting responses for each phase of this study.

DNAmix 2021 was conducted in 2012–2022, with the following dates for each phase:

• Registration: June 28, 2021-March 6, 2022
• P&P: July 9, 2021-March 10, 2022
• CSQ: September 2, 2021-March 17, 2022
• NoC: November 22, 2021-April 19, 2022
• ICSA: January 19, 2022-August 1, 2022

### 3.2 Participation

Participation was solicited via postings and announcements through relevant organizations, including the Organization of Scientific Area Committees for Forensic Science (OSAC), the Scientific Working Group on DNA Analysis Methods (SWGDAM), the Combined DNA Index System (CODIS) (at the 2021 National CODIS Conference), the American Academy of Forensic Sciences (AAFS), and the American Society of Crime Laboratory Directors (ASCLD). In order to encourage participation, we also presented at several conferences: the 2022 American Academy of Forensic Sciences conference, the American Society of Crime Laboratory Directors symposium, and the 2021 International Symposium on Human Identification (ISHI) conference.

Participation was open to all forensic laboratories that conduct DNA mixture interpretation as part of their SOPs. Non-U.S. laboratories were welcome to participate if they report interpretations in English. Laboratories were permitted to register more than one participant (termed “subunits”). It was left up to each laboratory's discretion whether to enroll multiple subunits, and which particular analysts that comprised each subunit (i.e., the identities of the individuals within a subunit were not known by the study team). Participants were advised that each subunit was required to complete each phase of the study completely independently from any other subunits. Participants agreed to use the same diligence in performing analyses as they used operationally in casework, including the lab's quality assurance process.

Each participant was required to complete an online informed consent form and registration form prior to study access (included as supplemental material). Participants were also asked to accept the terms and conditions required to participate in the study and complete a Registration and Configuration (RC) Questionnaire.

### 3.3 Questionnaire Development (Phases 1 & 2)

The first two phases of DNAmix 2021, and the particular focus of this paper, consisted of two multiple-choice questionnaires designed to collect information about laboratory procedures, case-specific decisions, and the nature of mixture casework. The specific questions and multiple-choice response categories were determined collaboratively with the DNAmix Working Group and DNAmix Advisory Group.

#### 3.3.1 Policies & Procedures Questionnaire Scope

The *P&P Questionnaire* was the first phase of the study and consisted of 93 total questions, intended to characterize the following for each laboratory:

• DNA workflow (quantification, amplification, capillary electrophoresis) details
• STR analysis software specifications
• Approach for assessing number of contributors (NoC)
• Criteria used for separating major and minor contributors
• Suitability assessments
• Non-statistical conclusions reported
• Statistical analyses conducted and reported
• Conditioning considerations
• Population databases utilized
• Probabilistic genotyping software policies (if applicable)
• Non-probabilistic genotyping software policies (if applicable)
• Reporting language used

Participants were informed that the responses from the *P&P Questionnaire* would be used to assess whether differences in SOPs across laboratories explain differences in interpretations, comparison, or statistical analysis of DNA mixtures.

#### 3.3.2 Casework Scenario Questionnaire Scope

The *CS Questionnaire* was the second phase of the study and consisted of 30 questions, intended to assess:

• Availability of a variety of case information
• Analysis options that vary at the case level
• Nature of mixture casework

Participants were informed that the responses from the *Casework Scenario Questionnaire* would be used to assess analysis procedures or decisions that may vary depending upon the case scenario and the nature of their mixture casework.

### 3.4 DNAmix Working Group and Advisory Group

In order to assure that the study design and details would be met with the approval of as wide a cross-section of the forensic DNA community as possible, we created two advisory groups to provide input—the DNAmix Working Group and the DNAmix Advisory Group—each consisting of a cross-section of forensic DNA analysts, academics, and researchers with DNA mixture analysis expertise, employed by a variety of agencies/institutions.

• The DNAmix Working Group was a group of volunteers with a range of expertise in DNA mixture analysis who were invited to provide input and guidance to the Bode/Noblis team on study design and details, including review of instructions, questions, and multiple-choice response categories for all four phases of the study. The information provided to working group members was limited so that they would be able to participate in the study; the information shared with the DNAmix Working Group was included in the instructions/FAQs provided to all participants to guarantee a level playing field. To avoid conflicts of interest, the DNAmix Working Group did not include anyone who works for any company that develops or sells PGS. The Working Group met (virtually in web conferences) from January-September 2021 (generally bi-weekly) and engaged in many email discussions. The Working Group included Jack Ballantyne (National Center for Forensic Science/University of Central Florida), Jen Breaux (Montgomery County Maryland), John Butler (NIST), Amber Carr (FBI Laboratory), Roger Frappier (center of Forensic Sciences, Toronto), Tim Goble (New York State Police), Bruce Heidebrecht (Maryland State Police), Kristy Kadash (Jefferson County (Colorado) Regional Crime Laboratory), Shawn Montpetit (San Diego Police Department), Steven Myers (California DOJ), Craig O'Connor (Office of Chief Medical Examiner of the City of New York), Robyn Ragsdale (Florida Department of Law Enforcement), Kristin Sasinouski (Bode Technology), and Charlotte Word (Private Consultant).The Working Group members were selected to include input from U.S. Federal, state, local, Canadian, private, and research perspectives. Working Group members included (but were not limited to) members of OSAC and SWGDAM.
• The DNAmix Advisory Group was created for discussions regarding mixture design decisions, detailed review of potential mixtures, and final approval of the mixtures used in the study. The Advisory Group was limited to members who were not eligible to participate in the study and were therefore considered “behind the curtain” for collaboration purposes. The Advisory Group was composed of the Bode/Noblis Study Team (Jonathan Davoren, Robert Bever, Austin Hicklin, Nicole Richetelli, Lauren Leone), the NIST Applied Genetics Group (Peter Vallone, Erica Romsos, Sarah Riman), and members of the DNAmix Working Group who were not eligible to participate in the study (Jack Ballantyne, John Butler, Charlotte Word). The Advisory Group met virtually as needed from February-November 2022.

*Note* the distinction that the Working Group was limited to issues related to the participant-facing portion of the study, whereas the Advisory Group focused on the design and selection of the mixtures. Eligible Working Group members were permitted to participate; Advisory Group members were not eligible to participate.

## Ethics Statements

The study protocol was reviewed by the Bode Technology Institutional Review Board (IRB) and met the NIJ requirements for human subjects research and privacy. An authorized representative from each participating laboratory completed an electronic informed consent form prior to participation in DNAmix 2021.

## CRediT authorship contribution statement

**Lauren M. Brinkac:** Methodology, Validation, Formal analysis, Investigation, Resources, Data curation, Writing – original draft, Writing – review & editing. **Nicole Richetelli:** Conceptualization, Methodology, Validation, Formal analysis, Investigation, Resources, Data curation, Writing – original draft, Writing – review & editing. **Jonathon M. Davoren:** Conceptualization, Methodology, Resources, Writing – review & editing, Funding acquisition. **Robert A. Bever:** Conceptualization, Methodology, Resources, Writing – review & editing, Funding acquisition. **R. Austin Hicklin:** Conceptualization, Methodology, Formal analysis, Investigation, Resources, Data curation, Writing – review & editing, Supervision, Project administration, Funding acquisition.

## Declaration of Competing Interest

The authors declare that they have no known competing financial interests or personal relationships that could have appeared to influence the work reported in this paper. This work was supported by the National Institute of Justice, Office of Justice Programs, U.S. Department of Justice Award No. 2020-R2-CX-0049. Lauren Brinkac, Nicole Richetelli, and Austin Hicklin are employees of Noblis, Inc. Jonathon Davoren and Robert Bever are employees of Bode Technology. To avoid conflicts of interest, Bode Technology analysts were not permitted to participate in the study.

## Data Availability

Laboratory Policies, Procedures, and Casework Scenario Decisions Relevant to DNA Mixture Interpretation: Data from the DNAmix 2021 Study (Original data) (OSF).

## Appendix Supplementary materials

Image, application 1

## Footnotes

1 This does not include 11 participants (from 9 laboratories) that only completed the registration/configuration form, and an additional 3 that did not complete registration. The “RC by Participant” tab in the P&P-CSQ dataset [1] includes all 190 participants that completed the registration/configuration form.

2 The country was ambiguous for one Non-U.S. laboratory.

3 *Note:* since the injection voltage and injection time questions were “check all that apply” conditioned on the CE instrument (and not on each other), we do not have the data to reliably report the intersection (as kV-s) between the two responses.

4 “Amp/CE Settings” refers to a specific combination of amplification kit, amplification cycles, volume of amplification reaction, CE instrument, and injection time and voltage.
