# Supplementary material for: DNAmix 2021: Laboratory policies, procedures, and casework scenarios summary and dataset
Source: Data Brief. 2023 Apr 14;48:109150. doi: 10.1016/j.dib.2023.109150 (PMC10147962; doi:10.1016/j.dib.2023.109150)
Supplement: Supplementary file 1 [file mmc1.pdf]

Inter-Laboratory Variation in Interpretation of DNA Mixtures Study

DNAmix 2021 Instructions — Registration and Questionnaires

Contents

1 Overview 1
2 Eligibility 2
3 Registration 2
3.1 Registering multiple participants from a laboratory 2
3.2 Online informed consent form 3
3.3 Online registration form 3
3.4 Online configuration questionnaire 3
4 Study Questionnaires (Phases 1 & 2) 3
4.1 Policies and Procedures Questionnaire 4
4.2 Casework Scenario Questionnaire 4
5 Anonymity and retrieving laboratory results 4
5.1 Anonymity 4
5.2 Retrieving laboratory results 5
Appendix A Informed Consent Form 6
Appendix B Registration Questions 9
Appendix C Configuration Questionnaire 10

1 Overview

This is a large-scale independent study being conducted to evaluate the extent of consistency and variation among forensic laboratories in interpretations, comparisons, and statistical analyses of DNA mixtures, and to assess the effects of numerous potential sources of variability. The study will evaluate the current state of the practice of DNA mixture casework and will not be restricted to specific products or statistical approaches. Noblis, Inc. and Bode Technology are conducting this study under National Institute of Justice (NIJ) grant award # 2020-R2-CX-0049, for the period 01/01/2021 to 12/31/2022.

This study will be composed of four phases:

1) *Policies and Procedures (P&P) Questionnaire* — Online questionnaire to assess laboratory policies and procedures relevant to DNA mixture interpretation (notably systems, types of statistics reported, and parameter settings used).

2) *Casework Scenario Questionnaire* — Online questionnaire to assess analysis procedures or decisions that may vary depending upon the case scenario and the nature of mixture casework conducted by the laboratory.

3) *Number of Contributors (NoC) Subtest* — Assessment of suitability and number of contributors for 14 mixture DNA samples, given electropherogram data.

4) *Interpretation, Comparison, and Statistical Analysis (ICSA) Subtest* — Interpretations, comparisons, and statistical analyses for 7 mixture DNA samples, given electropherogram data and reference profiles of assumed and potential contributors.

The DNAmix 2021 website (https://dnamix.edgeaws.noblis.org) will provide access to all online questionnaires and subtest electropherogram data (available for download) as well as a user interface for reporting responses for each phase of this study. This document provides instructions for registration and the first two phases (the *P&P Questionnaire* and *Casework Scenario Questionnaire*). A separate document will provide instructions for the *NoC* and *ICSA Subtests*.

2 Eligibility

Participation is open to all forensic laboratories that conduct DNA mixture interpretation as part of their standard operating procedures (SOPs). Non-U.S. laboratories are welcome to participate if they report the results of their interpretations, comparisons, and statistical analyses in English.

Participation in this study requires the participants to agree to use the same diligence in performing these analyses as used in operational casework, and to use their laboratory's SOPs in performing these analyses and conducting any quality assurance procedures required for the *NoC* and *ICSA* subtests.

Laboratories are encouraged to participate in the early phases even if they cannot commit to the later phases.

3 Registration

For the purposes of this study, participants are laboratories, not individuals. It is the discretion of participating laboratories to determine which analysts will be involved in the study, and the identities of the specific analysts will not be known to the DNAmix Study Team. Analysts involved must be qualified by the laboratory for operational mixture casework (not trainees). Laboratories will be permitted to register more than one participant, with each completing a separate registration; for additional details, please see *Section 3.1*.

Please go to the DNAmix 2021 study website (https://dnamix.edgeaws.noblis.org) to register for this study. The website is accessible using an ordinary web browser; there is no need to download or install any additional software or plugins. The website is compatible with the most recent releases of Google Chrome, Mozilla Firefox, Microsoft Edge, and Apple Safari. Using older versions may result in errors. Internet Explorer is not supported.

To register for this study, eligible laboratories must complete the following:

- Online consent form— see *Section 3.2*
- Online registration form— see *Section 3.3*
- Online configuration questionnaire— see *Section 3.4*

After completion of these items, you will use the email address that you provided along with the password you create during the registration process in order to access the DNAmix 2021 website. To enable the necessary security given that we are dealing with DNA samples, the study website requires two-factor authentication via text message to log in.

3.1 Registering multiple participants from a laboratory

Laboratories will be permitted to register more than one participant. Should a laboratory elect to submit multiple responses, each registered participant will be referred to hereafter as a "subunit." It is the laboratory's discretion whether to enroll subunits, and the particular analysts that comprise each subunit—the identities of specific individuals within a subunit will not be known to the DNAmix Study Team.

Each subunit must complete the entire registration process, including the consent form, registration form, and configurations questionnaire.

Each subunit will also be required to complete each phase of the study (the *Policies and Procedures questionnaire, Casework Scenario questionnaire*, *NoC subtest*, and *ICSA subtest)* **completely independently** from any other subunits within your laboratory or other laboratories.

If feasible, technical reviews and quality assurance procedures as outlined in the laboratory's SOPs should also be conducted for each subunit independently.

3.2 Online informed consent form

Participation in this study requires completion of an electronic informed consent form by a laboratory or subunit representative. The DNAmix 2021 website will provide access to the online consent form as part of the registration process. Please complete the required informed consent fields on the website. After completion of this form, we recommend that you print or save a PDF copy for your records.

The text for the online informed consent form is duplicated in this document in Appendix A.

3.3 Online registration form

You will then be prompted to complete the online registration form, which collects information about your laboratory and contact details for an administrative point of contact for your laboratory or subunit. Note that the cell phone number provided for the point of contact will be the one used for two-factor authentication when logging into the study website.

The text for the electronic registration form is duplicated in this document in Appendix B.

3.4 Online configuration questionnaire

The DNAmix 2021 website will then prompt you to complete the *Configuration Questionnaire*, which includes questions about the amplification kit(s) and capillary electrophoresis (CE) instrument(s) that your laboratory uses for DNA mixture casework.

Responses to the *Configuration Questionnaire* will be used by the study team in generating electropherograms for the *NoC subtest* and *ICSA subtest*. For each mixture sample and reference sample used in these subtests, we will prepare HID files for several popular combinations of “Amp/CE Settings”, which refers to a specific combination of:

- Amplification kit
- Amplification cycles
- Volume of amplification reaction
- CE instrument
- Injection time and voltage

The text for the online configuration questionnaire is duplicated in this document in Appendix C.

Note that in the Configuration Questionnaire, each question is submitted individually, and cannot be revised after submission.

4 Study Questionnaires (Phases 1 & 2)

Please review the DNAmix 2021 Glossary on the DNAmix 2021 website prior to beginning the *P&P Questionnaire* and the *Casework Scenarios Questionnaire* for details about the acronyms and terminology as specifically used in this study.

Both the *P&P Questionnaire* and the *Casework Scenarios Questionnaire* automatically save your progress as you complete each set of questions, and allow you to go back a revise your responses up until the point you submit at the end of each questionnaire.

The questionnaires do not need to be answered all at one time, but can be completed over time — IF you use the same browser and computer. NOTE: if you change computers or browsers the questionnaires will restart from the beginning.

4.1 Policies and Procedures Questionnaire

The *Policies and Procedures (P&P) Questionnaire* is the first phase of this study, and will be accessible via a link on the Participant Homepage of the DNAmix 2021 website. When the *P&P Questionnaire* initially becomes available, all registered participants will be notified via e-mail.

The *P&P Questionnaire* includes questions regarding your laboratory's:

- DNA workflow (quantification, amplification, capillary electrophoresis) details
- STR analysis software specifications
- Approach for assessing number of contributors (NoC)
- Criteria used for separating major and minor contributors
- Suitability assessments
- Non-statistical conclusions reported
- Statistical analyses conducted and reported
- Conditioning considerations
- Population databases utilized
- Probabilistic genotyping software policies (if applicable)
- Non-probabilistic genotyping software policies (if applicable)
- Reporting language used

Please answer all questions as completely and accurately as possible, based upon your laboratory's SOPs and any other policies and validated procedures utilized for DNA mixture casework.

The responses from this survey will be used by the study team to assess whether differences in SOPs across laboratories explain differences in interpretations, comparisons, or statistical analysis of DNA mixtures. In addition, the responses will be used to inform the design of and data selection for the subsequent phases of this study.

4.2 Casework Scenario Questionnaire

The *Casework Scenario Questionnaire* is the second phase of this study, and will be accessible via a link on the Participant Homepage of the DNAmix 2021 website. When the *Casework Scenario Questionnaire* initially becomes available, all registered participants will be notified via e-mail.

The *Casework Scenario Questionnaire* includes questions regarding:

- Availability of a variety of case information
- Analysis options that vary at the case level
- Nature of your mixture casework

The responses from this survey will be used by the study team to assess analysis procedures or decisions that may vary depending upon the case scenario and the nature of your mixture casework.

5 Anonymity and retrieving laboratory results

5.1 Anonymity

Study records and test results will be confidential. Laboratory and subunit information will be limited to what is necessary for administering the study. Reported results will not be linked to laboratories' identifying information: results will be reported using anonymized identifiers, without attribution to specific agencies. No reference will be made in oral or written reports, publications, or released datasets that could link your laboratory's name or

contact information to the study. Reported results will not be aggregated in a way that compromises confidentiality.

Responses will be kept separate from laboratory names and contact information during analyses of results. Confidentiality will be assured through multiple levels of data anonymization, data segregation, and controlled flow of information. Cross-references between laboratory information and the anonymized identifiers will be destroyed prior to the publication or public presentation of the results. Therefore, the identities of participating agencies will not be associated with the results at any point during analysis, and such association will not be possible subsequently, such as for discovery or FOIA requests.

5.2 Retrieving laboratory results

Results will be coded in a way that will allow participants to see their own anonymized results after completion of the study, if they choose to do so. After the submission of all samples (at the end of the *ICSA Subtest*), the software will give each participant the option to see its own Anonymous ID. This Anonymous ID will only be provided once: if a participant loses its Anonymous ID after it has been provided, that participant will not be able to see its results. The software will not record whether participants accessed their Anonymous IDs. If a laboratory includes multiple participating subunits, Anonymous IDs will be provided independently to each participating subunit: subunits within a laboratory will not be provided the Anonymous IDs for the other subunits. When the testing period is complete, but before public release of results, point-of-contact information will be deleted and the cross-references between laboratory information and anonymized identifiers will be deleted: after that point the study team will have no way to associate results with individual laboratories or look up/reassign anonymized identifiers. The final report will include an appendix with a table showing the results for each anonymized identifier, allowing those laboratories or subunits who chose to keep their anonymized identifiers to look up their results.

In some legal systems, knowledge of test results may create an obligation for the laboratory to disclose the test results in criminal, civil or regulatory proceedings. Prior to requesting Anonymous IDs, participating laboratories may wish to consult with their agency's counsel.

Appendix A Informed Consent Form

On the [DNAmix 2021 website], select "Register" and as part of the registration process you will be presented with the following informed consent form. Note that informed consent is completed online; this information is provided here as a reference.

Study Title

Inter-laboratory Variation in Interpretation of DNA Mixtures ("DNAmix 2021")

Sponsors

Noblis, Inc. and Bode Technology

Principal Investigators

R. Austin Hicklin, Ph.D. (Noblis) (703) 610-1995, hicklin@noblis.org
Jonathan Davoren, M.S. (Bode) (703) 317-7400, jonathan.davoren@bodetech.com

Additional Contact

Will Chapman, (703) 610-2983, william.chapman@noblis.org

Address

Noblis, 2002 Edmund Halley Drive, Reston, VA 20191 USA

Purpose

This study will be a large-scale, independent, rigorous empirical evaluation of the extent of variation among forensic laboratories in the statistical analysis and interpretation of electropherograms (EPGs) resulting from DNA mixtures. We plan to have between 50 and 150 laboratories taking part in this study. This study is being conducted under a grant from the National Institute of Justice (NIJ Grant #2020-R2-CX-0049).

Participation

Participation will be open to all forensic laboratories that conduct DNA mixture interpretation as part of their SOPs; non-U.S. laboratories are welcome to participate if they report interpretations in English. Participation in the study requires the participants to agree to use the same diligence in performing these analyses as used operationally in casework, and to use their laboratory's SOPs in performing these analyses.

Procedures

The study will consist of four subtests:

1. *Policies and Procedures Questionnaire — Online questionnaire to assess laboratory policies and standard operating procedures (SOPs) relevant to DNA mixture interpretation, interpretation or statistical software used, and parameter settings.*

2. *Scenario Questionnaire — Online questionnaire presenting a number of casework-derived scenarios (without DNA data), asking participants to assess how they would conduct analysis for each scenario.*

3. *Number of Contributors Subtest (NoC) — Assessment of suitability and number of contributors, given electropherogram data from DNA mixtures.*

4. *Statistical Analysis and Interpretation Subtest — Report of statistical results and categorical interpretations, given electropherogram data from DNA mixtures provided with DNA profiles of reference samples.*

All of the subtests will be administered online. Participants are encouraged to participate in the early subtests even if they do not participate in the later subtests.

If your laboratory expresses interest or agrees to participate, it will be sent a link to a website that will collect the laboratory name, point of contact, and email address for the point of contact. After this consent form is submitted, a link will be sent to the Policies and Procedures Questionnaire. Once a subtest of the study is completed, a link to the next subtest in the above list will be sent.

Confidentiality

Results will be confidential. No information about your laboratory will be released. No personally identifiable information (PII) will be released and results will not be attributed to participants. The research results will be published, but anonymity of participants will be maintained and results will not be associated with specific participants. Personally identifiable information (PII) will be used only for the purpose of conducting the study, and will not be used or released for other purposes. Your laboratory's study results will not be linked to its PII. No reference will be made in oral or written reports, publications, or released datasets that could link your laboratory's name to the study. A blind coding system will ensure anonymity. The subject ID numbers associated with your laboratory will be anonymized so that the analysis team will not be able to associate your laboratory's responses to any/all of the four subtests with your laboratory's name, email address, or laboratory representative. Cross-references between the subject IDs and the anonymized codes will be destroyed prior to the publication or public presentation of any results. Therefore, the identities of participating laboratories will not be associated with the results at any point during analysis, and such association will not be possible subsequently, such as for discovery.

Upon publication of the study results, your laboratory will be offered an opportunity to see its results using an Anonymous ID that will be provided when the last responses are submitted. This Anonymous ID will only be provided once: if your laboratory loses its Anonymous ID after it has been provided, your laboratory will not be able to see its results as the researchers will have no way of linking the Anonymous ID to your laboratory's name or contact information once the study is completed. The study team will have no way of knowing if your laboratory accessed its Anonymous ID. If your laboratory chooses to obtain its results, it is solely up to your laboratory's discretion as to whether or not to share its results with anyone, except as required by law.

The researchers will not disclose which laboratories did or did not take the test. In reporting results, results will be aggregated across multiple laboratories. Care will be taken so that the results are not aggregated in a way that compromises anonymity. The Principal Investigators and the Institutional Review Board (IRB) will be able to inspect confidential study-related records that identify your laboratory by name, which means that absolute confidentiality cannot be guaranteed.

Benefits

This study is for research purposes only. There is no direct benefit to your laboratory from participation in the study. The results of this study will be published in a peer-reviewed journal. The results of this research will provide the DNA analysis community with information regarding the accuracy, reproducibility, and repeatability of analyses produced in the discipline. This information can be used to improve analysis methodologies, training programs, and quality assurance measures. If the study indicates high performance by DNA analysis laboratories, this research may provide confidence in the legal community that DNA mixture analysis is reliable and provides added value to investigations and during courtroom proceedings. This research will inform future DNA mixture analysis studies.

Risks and Discomforts

No deception will be used in this study.

Analysts may experience physical fatigue (including eye strain) and mental fatigue if they perform analyses for an extended period of time. The scenarios in the Scenario Subtest are designed to resemble real casework and may be disturbing to some people.

In some legal systems, knowledge of your laboratory's test results may create an obligation for your laboratory to disclose those results in a criminal, civil or regulatory proceeding for which your laboratory is called to testify or provide evidence. If your laboratory chooses to access its own results, that information may then be under legal discovery when the laboratory acts as an expert witness in the future. If your laboratory elects to request its individual results, your laboratory is advised to consider first consulting with your laboratory's agency's counsel or counsel of your laboratory's choice.

The study team is not aware of any other risks, but there may be unknown risks associated with this study.

New Findings

Any new important information that is discovered during the study and which may influence your laboratory's willingness to continue participation in the study will be provided to your laboratory.

Alternatives

This research study is for research purposes only. Your laboratory is free to participate or not participate in this study. If your laboratory chooses not to participate, there will be no negative consequences.

Costs and Compensation for Participation

No charges will be billed to your laboratory or your agency for this study. Your laboratory will not be paid for its participation in this study.

Whom to Contact

If your laboratory has questions about the study, please contact the study staff listed on page one of this document. Please reference "DNAmix 2021" when contacting the Principal Investigators or study staff.

Refusal or Withdrawal of Participation

Participation in this study is voluntary. Participation in this research study is not mandatory; your laboratory may withdraw from the study for any reason without penalty. If your laboratory decides to participate, completion of the entire study is encouraged, but is not required. If your laboratory withdraws from the study before data collection is completed, you may notify the principal investigators if you wish for your laboratory's data to be destroyed. After the end of the data collection period (after the results from all participants have been collected), data will be anonymized and pooled and withdrawal of your laboratory's data will not be possible. Your laboratory's decision whether or not to participate in the study will not affect its current or future relations with the investigators. The investigators or the sponsor can stop your laboratory's participation at any time without your laboratory's consent.

Injury Statement

If anyone at your laboratory becomes ill or is injured while your laboratory is in the study, that individual is encouraged to get the medical care that s/he needs right away. If anyone at your laboratory is injured while engaged in the study or as a direct result of this study, your laboratory should contact the principal investigator at the number(s) provided on the first page of this form. Your laboratory will not lose any of its legal rights or release the sponsor, the Investigator, the study staff, or study site from liability for mistakes by signing this consent document.

Data Use Agreement

Due to human subjects research restrictions, the DNA profiles and mixtures included in this study shall not be used for any purpose other than this study: they shall not be stored, retained, or shared with anyone outside your laboratory; they shall not be used for research or internal validation studies; any copies or representations of the DNA profiles and mixtures shall be destroyed at the end of the study.

Consent to Take Part in this Research Study

I confirm that the purpose of the research, the study procedures and the possible risks and discomforts as well as potential benefits that I may experience have been explained to me. Alternatives to my laboratory's participation in the study also have been discussed. All of my questions have been answered. I have read this consent form. By agreeing to this informed consent form, my laboratory agrees that it will not save, copy, or redistribute any of the files included in the study.

By selecting "I consent to taking part in this study" below, as an authorized representative of my laboratory, I freely give consent for my laboratory to take part in this study.

Appendix B Registration Questions

On the DNAmix 2021 website, as part of the registration process you will be asked for the following information after the informed consent form. Note that registration is completed online; this information is provided here as a reference.

Please read "DNAmix 2021 — Overview and Registration Instructions" (available on the DNAmix 2021 website) prior to completing this form. That document provides an overview of the study and details on eligibility, registration, and anonymity of results.

Please provide an administrative point of contact for this participating laboratory. Alternatively, if you are participating as part of a subunit, please provide the point of contact for your subunit. This information will only be used to administer the study. This email address will be used to log into the study website to access data and provide responses. This cell phone number will be the one used for two-factor authentication when logging into the study website: to log into the study website, a code will be texted to this cell phone that must be entered in the website to proceed.

- *Point of Contact First Name*
- *Point of Contact Last Name*
- *Point of Contact Email Address*
- *Point of Contact Cell Phone Number (Must be able to receive text messages. Do not include dashes or spaces. US phone numbers must start with "+1"; non-US numbers start with "+" and country code.)*
- *Password (Must be at least 12 characters, and include at least one each of [uppercase letters, lowercase letters, digits, and symbols])*
- *Confirm Password*

Appendix C Configuration Questionnaire

On the [DNAmix 2021 website](), as part of the registration process you will be asked for the following information after the registration form. Note that registration is completed online; this information is provided here as a reference.

Note that in the Configuration Questionnaire, each question is submitted individually, and cannot be revised after submission.

The purpose of these questions is to gather information from participating laboratories about the type of laboratory, and about the amplification kit(s) and capillary electrophoresis (CE) instrument(s) that your laboratory uses for DNA mixture casework. The study team will use this information to select the settings used in creating mixtures and resulting electropherograms that will be used in the later phases of this study (NoC subtest and ICSA subtest). For each mixture, we will create electropherograms/.HID files to accommodate as many participants as possible.

When filling out this questionnaire, please respond only regarding STR identity testing (not mitochondrial, paternity, or familial testing). Respond only regarding DNA mixtures (not single-source samples).

Names of commercial manufacturers are included for systems that are frequently used in laboratories; inclusion does not imply endorsement by the study team.

- *Does your laboratory conduct DNA mixture analysis and report the results in English?*
  - *Yes*
  - *No (Not eligible for study)*
- *By participating in this study, you affirm that*

  1) You will use the same diligence in performing these analyses as used in casework.
  2) Your responses to all surveys are accurate.
  3) You will not share or redistribute the electropherograms obtained during this study.
  4) At the completion of the study, you will destroy all electropherograms obtained as a part of this study.
  - *I agree on behalf of my laboratory, and my laboratory will abide by all four of these conditions*
  - *I do not agree and understand that we cannot participate (Not eligible for study)*
- *Please provide your laboratory name*
- *What type of laboratory do you represent?*
  - *U.S. Local laboratory*
  - *U.S. State laboratory*
  - *U.S. Federal laboratory*
  - *U.S. Private laboratory*
  - *Non-U.S. Local laboratory*
  - *Non-U.S. State/Provincial laboratory*
  - *Non-U.S. Federal/National laboratory*
  - *Non-U.S. Private laboratory*
- *How many DNA analysts conduct mixture analysis in your laboratory? (For the purposes of this study, a DNA analyst is an individual who has completed training and is qualified by your laboratory to conduct independent operational mixture casework.)*
  - *1*
  - *2-10*
  - *11-25*
  - *26-50*
  - *51+*
- *Please indicate all autosomal STR amplification kit(s) that are validated and used in your laboratory for DNA mixtures (check all that apply). For each, please specify all validated amplification cycle settings specified by your SOPs (separate multiple values with commas).*

- *Applied Biosystems AmpFLSTR Identifiler (specify amp cycles)*
- *Applied Biosystems AmpFLSTR Identifiler Plus (specify amp cycles)*
- *Applied Biosystems AmpFLSTR Profiler (specify amp cycles)*
- *Applied Biosystems AmpFLSTR Profiler Plus (specify amp cycles)*
- *Applied Biosystems GlobalFiler (specify amp cycles)*
- *Promega Powerplex 16 (specify amp cycles)*
- *Promega Powerplex 16 HS (specify amp cycles)*
- *Promega Powerplex Fusion 5C (specify amp cycles)*
- *Promega Powerplex Fusion 6C (specify amp cycles)*
- *Qiagen Investigator 24plex (specify amp cycles)*
- *Other (specify name and version)*

- *Please indicate all CE instrument(s) are validated and used for mixture casework in your laboratory (check all that apply). For each, please specify all validated voltage and injection time settings specified by your SOPs (separate multiple values with commas).*
  - *Applied Biosystems 3100 (specify voltage(s) and injection time(s))*
  - *Applied Biosystems 3130 (specify voltage(s) and injection time(s))*
  - *Applied Biosystems 3130xl (specify voltage(s) and injection time(s))*
  - *Applied Biosystems 3500 (specify voltage(s) and injection time(s))*
  - *Applied Biosystems 3500xl (specify voltage(s) and injection time(s))*
  - *Applied Biosystems 3700 (specify voltage(s) and injection time(s))*
  - *Applied Biosystems 3730 (specify voltage(s) and injection time(s))*
  - *Promega Spectrum (specify voltage(s) and injection time(s))*
  - *Other (specify name)*

Inter-Laboratory Variation in Interpretation of DNA Mixtures Study:

Number of Contributors (NoC) Subtest Instructions

Contents

1 Overview 1
2 Mixture Configuration Selection 2
3 NoC Packets: DNA Mixture Profiles 2
3.1 Amp/CE Settings........ 2
3.2 Preparation of DNA Mixture Profiles 3
3.3 Data Provided........ 3
4 NoC Subtest Questions 4

1 Overview

The *Number of Contributors (NoC) Subtest* is the third phase of the DNAmix 2021 study. In this subtest you will be assigned 12 *NoC Packets*, each containing a DNA mixture profile. All DNA mixture profiles will be provided as electropherograms (HID files). Each electropherogram you are assigned will be prepared using a specified combination of *Amp/CE Settings*[*] you select in *Mixture Configuration Selection,* which must be completed prior to the *NoC Subtest* (see Section 2 for additional details).

For each *NoC Packet*, participants will be asked to provide assessments of suitability and number of contributors.

The *Number of Contributors (NoC) Subtest* will be accessible via a link on the Participant Homepage of the DNAmix 2021 website. When the *NoC Subtest* initially becomes available, all registered participants will be notified via e-mail. Prior to the launch of the *NoC Subtest,* a "NoC Beta Test" will be temporarily available, which will consist of one comparison packet for review. Although the *NoC Beta Test* is not required, participants are highly encouraged to complete it as practice and are welcomed to provide feedback for improving the *NoC Subtest* (e.g., clarity of questions, functionality of software, etc.). Once the *NoC Subtest* becomes available, the *NoC Beta Test* will be removed from the website.

Conduct your assessments of each DNA mixture profile (HID file) and respond to each of these questions based upon the policies and validated procedures in your Standard Operating Procedures (SOPs), using the same considerations and diligence that you would employ for operational casework samples.

Please review the "DNAmix2021 — Glossary" prior to beginning the *NoC Subtest* for details about the acronyms and terminology as specifically used in this study.

The *P&P Questionnaire* and the *Casework Scenario Questionnaire* (Phases 1 and 2 of the DNAmix 2021 study) may be completed at any time throughout the study period and need not be submitted in order to complete the *NoC Subtest*. However, participating laboratories must complete and submit these questionnaires prior to the end of the study in order to retrieve their study results.

[*] *"Amp/CE Settings" refers to a specific combination of amplification kit, amplification cycles, volume of amplification reaction, CE instrument, and injection time and voltage*

2 Mixture Configuration Selection

Mixture Configuration Selection is a two-question online survey accessed from the DNAmix 2021 website conducted for participants to:

- Indicate if you will participate in the NoC and/or ICSA Subtests
- Select the Amp/CE settings used to prepare the mixtures that will be assigned to you in the NoC/ ICSA Subtests
- Indicate how the selected Amp/CE Settings compare to your SOPs

Before you begin the *NoC Subtest*, you must complete *Mixture Configuration Selection*. The study team will use this information to assign mixtures to participants, and the DNAmix website software will use the information to determine which questions and response options are presented in the NoC and ICSA Subtests.

Mixture Configuration Selection includes the following questions:

- *Select one of the following Amp/CE Settings that you will use to participate in the NoC and/or ICSA Subtests of this study:*
    - 6C29: Promega PowerPlex Fusion 6C@29 cycles; Amp volume 25µL; ABI 3500xl injection at 1.2kV for 24 seconds (equivalent to ABI 3500 for 15 seconds)
    - GF28: Applied Biosystems GlobalFiler@28 cycles; Amp volume 25µL; ABI 3500xl injection at 1.2kV for 24 seconds (equivalent to ABI 3500 for 15 seconds)
    - GF29: Applied Biosystems GlobalFiler@29 cycles; Amp volume 25µL; ABI 3500xl injection at 1.2kV for 24 seconds (equivalent to ABI 3500 for 15 seconds)
    - ID28: Applied Biosystems AmpFLSTR Identifiler Plus@28 cycles; Amp volume 15µL; ABI 3500xl injection at 1.2kV for 12 seconds (equivalent to ABI 3500 for 7.5 seconds)
    - None of the above (We will not participate in the NoC or ICSA Subtests)
- *[if not "none of the above"] Please indicate how the selected Amp/CE Settings compare to your SOPs:*
    - This corresponds exactly to our lab's validated settings—we can use these settings for NoC and ICSA
    - This is equivalent to our lab's settings; this differs in details we consider minor or inconsequential (such as injection time of 15 vs 16 seconds)—we can use these settings for NoC and ICSA
    - This differs from our lab's validated settings, but we are willing to participate using these settings in both NoC and ICSA (Note: these results will be analyzed separately during analysis)
    - This differs from our lab's validated settings; we are willing to participate using these settings in NoC, but not in ICSA (Note: these results will be analyzed separately during analysis)

3 NoC Packets: DNA Mixture Profiles

In the *NoC Subtest* you will be assigned a total of 12 *NoC Packets*. Each *NoC Packet* includes one DNA mixture profile, positive and negative controls, and allelic ladders. No case information will be provided. All DNA mixture profiles are electropherograms, provided to participants as HID files.

You will have access to only *NoC Packet* at a time: to avoid the possibility of administrative errors or misunderstandings, you must submit your responses for a DNA mixture profile before downloading the next DNA mixture profile.

3.1 Amp/CE Settings

In order to represent the SOPs of as many participating laboratories as feasible, electropherograms were prepared using four combinations of "Amp/CE Settings" (which refers to a specific combination of amplification kit, amplification cycles, volume of amplification reaction, CE instrument, and injection time and voltage). The combinations of Amp/CE Settings that have been implemented were the four most commonly-used Amp/CE

settings selected by registered participants,† using the abbreviations listed in *Section 2 (Mixture Configuration Selection)* [6C29, GF28, GF29, or ID28].

You will be assigned mixtures that were prepared using the Amp/CE setting option that you chose during *Mixture Configuration Selection*, prior to the *NoC Subtest*.

3.2 Preparation of DNA Mixture Profiles

The DNA used to create the mixture profiles for this study came from various sources, including buccal, blood, and tissue samples. There were no simulated/contrived profiles; all DNA profiles in this study are from real people. DNA samples were extracted prior to mixing.

Mixtures were quantified using ABI Quantifiler Trio on an ABI 7500 real-time PCR instrument. The mixture quantification results (including the total amount of DNA amplified, amount of male DNA, and degradation index) will be included with the mixtures in the *NoC Subtest*.

Various volumes of DNA were pipetted into a single tube to make a large mixture stock. That stock was then aliquoted and amplified in each of the four amp kits (see "Amp/CE settings" above for amplification volumes and cycles). The ABI 9700 thermocycler was used for amplification, using the specific Amp/CE settings and other standard manufacturer recommended settings. The ABI 3500xl was used for capillary electrophoresis (CE), using injection time and voltage settings specified in the Amp/CE Settings (see above); settings for run time, run voltage, capillary length, polymer type, etc. use the default settings specified for each amp kit.

GeneMapper (v1.5; incorporated into ABI 3500xl) was used to create HID files. We are not providing PDFs (images) of the electropherograms because creating such PDFs implements decisions regarding the analytical threshold (AT) value and the utilization of stutter filters, and we want all such decisions to be made by the participants.

Every effort was made with respect to quality assurance in creating these mixtures. Note that in some cases there may be artifacts (such as pull-up) present, as may be found in ordinary casework — please review the controls provided.

3.3 Data Provided

Each *NoC Packet* is numbered (NoC_01 through NoC_99, shown as "NoC_XX" in the table below). Participants are not necessarily assigned the same packets, and the order of assignments varies among participants.

Each *NoC Packet* is specific to the Amp/CE Settings previously selected by participants (shown as "YYYY" in the table below), using the abbreviations listed in *Section 2 (Mixture Configuration Selection)* [6C29, GF28, GF29, or ID28].

Each *NoC Packet* is contained in a Zip file, downloaded from the DNAmix 2021 website (https://dnamix.edgeaws.noblis.org/). Each *NoC Packet* includes the following files:

All Packets	1 DNA mixture profile (HID file)	NoC_XX_YYYY_Mixture.hid
	Amp/CE Settings used to create the electropherograms	AmpCESettings_YYYY.pdf
	Quantitation data for the mixture	NoC_XX_QuantResults.pdf
	2 Ladders	NoC_XX_YYYY_Ladder1.hid<br>NoC_XX_YYYY_Ladder2.hid

† *Registered participants were contacted by email and were given a deadline of 23 August 2021 to indicate preferences for Amp/CE settings.*

	Positive and Negative controls	NoC_XX_YYYY_Pos.hid<br>NoC_XX_YYYY_Neg.hid

In a few cases the positive or negative controls were re-injected, in which case they are in a subdirectory (named POS or NEG) with the associated ladders.

4 NoC Subtest Questions

On the DNAmix 2021 website, you will be asked to answer the following questions for ***each*** of the 12 DNA mixtures that you are assigned in the *NoC Subtest*. The *NoC Subtest* is completed online; this information is provided here as a reference.

As a quality assurance measure, the website will display an image of the electropherogram for the first several loci in the DNA mixture profile for the assigned *NoC Packet*. Please ensure that you are submitting your responses for the given mixture profile.

After downloading the packet, we recommend clicking HOME in the main menu bar (at the top of the DNAmix webpage) and returning to the NoC Subtest screen only when you are ready to enter your responses. (Some users have timeout issues if this window is left open.)

Please enter and submit all responses for the assigned *NoC Packet* in one session. You will not be able to save your responses and return to complete response entry within a single *NoC Packet*; if you exit prior to submitting, you will be required to re-enter all of your responses for the *NoC Packet*.

Note for each mixture profile assessed in the NoC Subtest, you will be asked to review and confirm your responses prior to submission. After submission, your responses are considered final and cannot be changed.

Packet Assignment Details

1. Please re-enter the Participant ID shown at the top of the page (Dxxxx):_____

   Note: this information will be used for quality assurance purposes only. The Participant ID (a 5 character alpha-numeric string starting with D) is located at the top right of the Number of Contributors (NoC) Page (right about the electropherogram preview image).

2. Please double-check the NoC Packet number: verify that the number of the HID mixture file you are assessing is the same as shown at the top of this page. Please enter that NoC Packet number here (For example, in the NoC Beta Test, you would enter the following NoC packet number: 99): _____

   The NoC Packet number is located in the HID filename, in the electropherogram preview image (located at the top of the NoC Subtest page of the DNAmix 2021 website), and embedded within the electropherogram data. You do not need to enter the "NoC_" portion; please only enter the two digit NoC Packet number.

Settings

3. Did you use an analytical threshold (AT) for this mixture?

   In other words, was there a minimum RFU value (either globally or per dye channel) to delineate signal (above the threshold) from noise (below the threshold)? This analytical threshold may have been utilized explicitly (by comparing measured RFU values to the threshold value(s)) or via general evaluation ("eye-balling" the data and comparing to the threshold value(s)).

   3.a Yes, I used a single AT (Please specify:_____)
   3.b Yes, but my ATs varied by dye channel

3.c No

4. Did you use a stochastic threshold (ST) for this mixture?

> *In other words, was there a minimum RFU value (either globally or per dye channel) to delineate peaks (above the threshold) from potential artifacts or stochastic effects (below the threshold)? This stochastic threshold may have been utilized explicitly (by comparing measured RFU values to the threshold value(s)) or via general evaluation ("eye-balling" the data and comparing to the threshold value(s)).*

4.a Yes, I used a single ST (Please specify:_____)
4.b Yes, but my STs varied by dye channel
4.c No

Replicate Amplifications

> *The next two questions both ask whether you would have conducted replicate amplifications if you had received this sample in actual casework, but please note the differences. Question 5 is regarding replicate amps based on the amount of DNA available: if only a small amount of DNA was available, would you have amplified all the DNA or divided it and done multiple amps? Question 6 is regarding replicate amps based on the review of the electropherogram: if additional DNA were available, after reviewing this EPG would you conduct another amp?*

5. If you received this DNA mixture sample in casework and the total quantity of DNA available was the amount specified in the quantitation data, would you have divided the sample and conducted multiple replicate amplifications?

> *In other words, given the DNA quantity alone (not based upon review of the electropherogram), would you have amplified the entire sample (as was done here), or would you have instead done 2 or more amplifications each using a part of the total sample? Assume that all DNA available in the entire (wet) sample was used here and there will not be additional DNA to permit a subsequent amplification after CE.*

5.a We do not ever conduct replicate amplifications in my laboratory (per our SOPs)
5.b No: we would not have done replicate amplifications in this case (but we do in some cases)
5.c Yes: we would do 2 replicate amplifications, each with 1/2 of the total amount, and increase sensitivity by adding 1 cycle
5.d Yes: we would do 3 replicate amplifications, each with 1/3 of the total amount, and increase sensitivity by adding 1 cycle
5.e Yes: we would do 3 replicate amplifications, each with 1/3 of the total amount, and increase sensitivity by adding 2 cycles
5.f Other (Please explain:_____)

6. If there was sufficient DNA remaining for an additional amplification, would you do another amplification (re-amp) after seeing this mixture profile?

> *In other words, if sufficient DNA remained would you conduct another amplification based upon the mixture profile/electropherogram provided in this NoC packet (e.g., to verify alleles, observe stochastic effects, etc.)?*

6.a No: we are not permitted to re-amp per our SOPs
6.b No: we would interpret this profile
6.c Yes: we would re-amp using more DNA
6.d Yes: we would re-amp using less DNA
6.e Yes: we would re-amp using the same amount of DNA

Suitability

7. Is this DNA mixture profile suitable for comparison and/or statistical analysis?

> *In other words, did you determine that this DNA mixture profile can appropriately be used to conduct comparisons (i.e., comparison of the mixture to reference profiles of POIs, victims, consensual partners, and/or expected contributors) and/or statistical analyses (i.e., compute an LR, RMP, or CPI/CPE with respect to a POI)?*

7.a Yes (for the entire mixture and all contributors)
7.b Yes, but only for a subset of the contributors (e.g., major(s))
7.c Yes, but only for a subset of loci
7.d Yes, but only for a subset of loci, and only for a subset of the contributors
7.e No

8. *[If the mixture is NOT suitable for comparison and/or statistical analysis]* Why is this profile unsuitable for comparison and statistical analysis? (check all that apply; check at least one)

> *In other words, if you indicated "No" in the previous question, what factor(s) informed your determination? Please select all factors that you considered in your determination that the DNA mixture profile was not suitable for comparison/statistical analysis.*

8.a Not enough alleles or loci suitable for analysis
8.b DNA template levels too low overall
8.c Sample too degraded
8.d Sample too inhibited
8.e Too many contributors
8.f Too much uncertainty in the number of contributors
8.g Mixture proportions/contributor ratios
8.h Other (Please specify:_____)

Number of Contributors *[Only shown if the mixture is suitable for comparison and/or statistical analysis]*

9. How would you report the number of contributors in this profile?

> *In other words, how would you report the number of contributors to this DNA mixture profile if you encountered this mixture in casework? Would you be able to assess the number of contributors given this DNA mixture profile? If so, would you report an exact/single estimate of number of contributors (e.g., 3 contributors) or would you report a range of possible numbers of contributors (e.g., 3-4 contributors or minimum of 3 contributors/maximum of 4 contributors) or would you report a minimum number of contributors (e.g., at least 3 contributors)?*

9.a I would report an exact number of contributors
9.b I would report a range of possible numbers of contributors
9.c I would report a minimum number of contributors
9.d The levels (overall quantity and/or peak heights) are not sufficient to determine the number of contributors [Go to Additional Comments]
9.e *The mixture is too complex to determine the number of contributors* [Go to Additional Comments]

9.1 Provide your estimate of NoC.

> *Note: you will only see the version of question 8.1 that is associated with your response to question 8 above. In other words, you will only see one of the three options in the question text below.*

– *[if selected 8a: Exact NoC]* Select your estimate of the number of contributors:

> *In other words, select your single estimate for the number of contributors to this DNA mixture profile.*

– *[if selected 8b: Range of NoC]* Select your estimate of the range of possible numbers of contributors. (For example, if you estimate that there are 3-5 possible contributors to this mixture profile, you must select 3, 4, and 5): (check all that apply; select at least two)

> *In other words, select all possible numbers of contributors included in your estimated range for this DNA mixture profile, which must include at least two options based upon your response to Q8 that you would report the NoC for this profile using a range of possible numbers of contributors.*

– *[if selected 8c: Exact NoC]* Select your estimate of the minimum number of contributors:

> *In other words, select your single estimate for the minimum number of contributors to this DNA mixture profile.*

9.1-a *At least 1 contributor*
9.1-b *At least 2 contributors*
9.1-c *At least 3 contributors*
9.1-d *At least 4 contributors*
9.1-e *At least 5 contributors*
9.1-f *At least 6 contributors*
9.1-g *At least 7 contributors*
9.1-h *At least 8 or more contributors*

10. What were the PRIMARY loci used as the basis for determining the number of contributors? In other words, indicate the loci that were most informative or most helpful. (check all that apply; select at least one)

> *Note: you will only see the set of loci included in the amplification kit that you previously selected in Mixture Configuration Selection.*
>
> *Names of commercial manufacturers are included for the systems that are the most frequently used by registered participants; inclusion does not imply endorsement by the study team.*

Applied Biosystems GlobalFiler (display order)	Promega PowerPlex Fusion 6C (display order)	Applied Biosystems AmpFLSTR Identifiler Plus (display order)
D3S1358	Amel	D8S1179
vWA	D3S1358	D21S11
D165539	D1S1656	D7S820
CSF1PO	D2S441	CSF1PO
TPOX	D10S1248	D3S1358
Y indel	D13S317	TH01
Amel	Penta E	D13S317
D8S1179	D16S539	D16S539
D21S11	D18S51	D2S1338
D18551	D2S1138	D19S433
DYS391	CSF1PO	vWA
D2S441	Penta D	TPOX
D19S433	TH01	D18S51
TH01	vWA	Amel
FGA	D21S11	D5S818
D22S1045	D7S820	FGA
D5S818	D5S818	
D13S317	TPOX	
D7S820	D8S1179	
SE33	D12S391	
D10S1248	D19S433	
D1S1656	SE33	
D12S391	D22S1045	
D2S1338	DYS391	
	FGA	
	DYS576	
	DYS570	

11. Which factors affected your assessment of number of contributors? (check all that apply; select at least one)

In other words, what factors did you consider when estimating the number of contributors for this DNA mixture sample? Please select all factors that informed your determination.

11.a Discriminating potential/variability of loci (or allele frequency)
11.b Expected stutter ratios
11.c Information below the analytical threshold
11.d Maximum Allele Count (MAC) per locus
11.e Overall level of data (peak heights in relation to laboratory validated thresholds)
11.f Peak heights (RFU)
11.g Peak morphology (e.g., CE resolution; unresolved microvariants; peak shouldering)
11.h Presence of degradation
11.i Presence of inhibition
11.j Quantitation data
11.k Relative peak heights (peak height ratios and possible shared/stacked alleles)
11.l Sex determining markers
11.m Total allele count in sample
11.n Other (Please specify:______)

12. Are you able to identify any major contributors?

In other words, would you consider one (or more) contributors to be major contributors according to the criteria outlined in your SOPs (e.g., based upon peak height ratios or RFU percentages). This separation of

> *major contributor(s) may have been conducted explicitly (by computing peak height ratios/RFU percentages and comparing to a threshold, such as a 3:1 peak height ratio or 70% of the total RFUs) or via general evaluation (distinguished visually, without calculation). If your SOPs do not permit you to differentiate between major and minor contributors, please indicate as such.*

12.a There are no contributors I would consider majors
12.b There is one major contributor
12.c There are two or more major contributors
12.d We do not differentiate between major and minor contributors

13. Did you use any software tool to assist in assessing the number of contributors?

> *In other words, please indicate how you assessed number of contributors for this DNA mixture profile. If you used a combination of manual assessment and software, please select the option which most informed your assessment.*

13.a No, I assessed the number of contributors manually
13.b Yes, I used NOCIt
13.c Yes, I used PACE
13.d Yes, I used FaSTR/STRmix
13.e Yes, I used an internally developed tool
13.f Yes, I used another commercial or open-source tool (Please specify:_____)

Additional Comments

14. Additional comments: Please provide a comment ONLY if there is an issue or a limitation for this NoC packet that you could not adequately address using any of your responses above. (Please limit your responses to 75 words or less.)

DNAmix 2021 — Inter-Laboratory Variation in Interpretation of DNA Mixtures Study

Interpretation, Comparison, and Statistical Analysis (ICSA) Subtest Instructions

Contents

1 Overview 1
2 ICSA Settings Selection 2
3 Comparison Packets: DNA Mixture Profiles and Reference Profiles 3
3.1 Amp/CE Settings 4
3.2 Preparation of DNA Mixture Profiles 4
3.3 Data Provided 5
4 Reporting Statistical Analysis Results 6
5 ICSA Subtest Questions 7

1 Overview

The *Interpretation, Configuration, and Statistical Analysis (ICSA) Subtest* is the fourth and final phase of the DNAmix 2021 study. In this subtest you will be assigned 8 *Comparison Packets* (each containing one DNA mixture profile and one or more reference profiles). All DNA profiles (mixture and reference) will be provided as electropherograms (HID file format). Each electropherogram you are assigned will be prepared using the specified combination of *Amp/CE Settings* you selected in *Mixture Configuration Selection*.

For each *Comparison Packet*, participants will be asked to provide categorical comparison conclusions and statistical analysis responses for two scenarios:

- ***General case*** — The categorical conclusion and supporting statistics that would be provided if there is no reason to assume that a close relative of the person of interest may be a suspect. Participants will also be asked to optionally provide their supporting statistics computed using only the 20 CODIS core loci for the general case. For participants who report likelihood ratios, this is expressed as LR=(Hp = the person of interest is a contributor to the mixture / Hd = an ***unrelated individual*** is a contributor to the mixture).
- ***Sibling case*** — (Optional) The categorical conclusion and supporting statistics that would be provided if a sibling (but not an identical twin) of the person of interest is also a suspect, but for whom a DNA profile is not available. For participants who report likelihood ratios, this is expressed as LR=(Hp = the person of interest is a contributor to the mixture / Hd = a ***sibling of the POI*** is a contributor to the mixture and the POI is NOT a contributor to the mixture). Participants will only report sibling case categorical conclusions and statistics if their SOPs allow such in casework.

For each *Comparison Packet*, participants will also be asked to provide assessments of suitability and number of contributors (as in the *NoC Subtest*).

The *ICSA Subtest* will be accessible via a link on the Participant Homepage of the DNAmix 2021 website. When the *ICSA Subtest* initially becomes available, all registered participants will be notified via e-mail. Prior to the launch of the *ICSA Subtest,* an "ICSA Beta Test" will be temporarily available, which will consist of one comparison packet for review. Although the *ICSA Beta Test* is not required, participants are highly encouraged to complete it as practice and are welcomed to provide feedback for improving the *ICSA Subtest* (e.g., clarity of questions, functionality of software, etc.). Once the *ICSA Subtest* becomes available, the *ICSA Beta Test* will be removed from the website.

Conduct your interpretations, comparisons, and statistical evaluations of each DNA mixture profile and respond to each of these questions based upon the policies and validated procedures in your Standard Operating Procedures (SOPs), using the same considerations and diligence that you would employ for

operational casework samples. Your responses should go through technical review/quality assurance as specified by your laboratory's SOPs.

Please review the "DNAmix2021 — Glossary" prior to beginning the *ICSA Subtest* for details about the acronyms and terminology as specifically used in this study.

Names of commercial manufacturers are included for the systems that are frequently used in laboratories; inclusion does not imply endorsement by the study team.

2 ICSA Settings Selection

In the *ICSA Subtest*, you will report your statistical responses for the population databases you use operationally, for each of the following populations:

- Black/African American
- White/Caucasian
- Combined Asian (or East Asian or Chinese)
- Combined Hispanic (or SW Hispanic or Mexican-American)
- Overall Combined

For example, if in your casework you use a Black/African American population database and a White/Caucasian population dataset (but do not use any of the others), you will report statistical responses using only those two tables.

Before you start the *ICSA Subtest*, you must indicate whether you use each type of population database and which specific databases you use in *ICSA Settings Selection*, which is a brief online questionnaire accessed from the DNAmix 2021 website.

ICSA Settings Selection includes the following questions:

1. Please indicate which Black/African American population database you use in your casework, and will use in this study (also described as "Combined African American, Bahamian, and Jamaican" or "Black African/Caribbean"):
   - 1.a *FBI STR Population Data: African American Allele Frequencies (includes African American, Bahamian, and Jamaican) (Expanded 2015 STR Allele Frequency Table)*[*]
   - 1.b *NIST 1036 Revised AfAm (2017)*[†]
   - 1.c *Other (Please specify: ______)*
   - 1.d *We do not use a Black/African American population database*

   1.1 *[if you use a Black/African American population database]* Please specify the value of theta that you use for the Black/African American population database that you use in casework, and will use in this study (Note: negative values and values greater than 0.2 will be regarded as typographical errors): ______

2. Please indicate which White/Caucasian population database you use in your casework, and will use in this study:
   - 2.a *FBI STR Population Data: Caucasian Allele Frequencies (Expanded 2015 STR Allele Frequency Table)*
   - 2.b *NIST 1036 Revised Cauc (2017)*
   - 2.c *Other (Please specify: ______)*
   - 2.d *We do not use a White/Caucasian population database*

---

[*] *Current (2015) FBI population databases can be found at https://www.fsigenetics.com/article/S1872-4973(16)30142-9/fulltext (in the supplemental table https://www.fsigenetics.com/cms/10.1016/j.fsigen.2016.07.022/attachment/bdb74544-07b8-4a23-ad28-46b3362de740/mmc1.xlsx).*

[†] *Current (2017) NIST population databases can be found at https://strbase.nist.gov/NISTpop.htm .*

2.1 *[if you use a White/Caucasian population database]* Please specify the value of theta that you use for the White/Caucasian population database that you use in casework, and will use in this study (Note: negative values and values greater than 0.2 will be regarded as typographical errors): _____

3. Please indicate which Combined Asian population database you use in your casework, and will use in this study. If you do not use a Combined Asian population database in casework, please indicate the East Asian or Chinese population database you use in your casework, and will use in this study (if applicable):
   - *3.a NIST 1036 Revised Asian (2017)*
   - *3.b Other Combined Asian population database (Please specify: _____)*
   - *3.c Other East Asian or Chinese population database (Please specify: _____)*
   - *3.d We do not use a Combined Asian, East Asian, or Chinese population database*

3.1 *[if you use a Combined Asian/ East Asian/Chinese population database]* Please specify the value of theta that you use for the Combined Asian, East Asian, or Chinese population database that you use in casework, and will use in this study (Note: negative values and values greater than 0.2 will be regarded as typographical errors): _____

4. Please indicate which Combined Hispanic population database you use in your casework, and will use in this study. If you do not use a Combined Hispanic population database in casework, please indicate the Southwestern Hispanic or Mexican-American population database you use in your casework, and will use in this study (if applicable):
   - *4.a FBI STR Population Data: Southwest Hispanic Allele Frequencies (Expanded 2015 STR Allele Frequency Table)*
   - *4.b NIST 1036 Revised Hispanic (2017)*
   - *4.c Other Combined Hispanic population database (Please specify: _____)*
   - *4.d Other SW Hispanic or Mexican-American population database (Please specify: _____)*
   - *4.e We do not use a Combined Hispanic, SW Hispanic, or Mexican-American population database*

4.1 *[if you use a Combined Hispanic/SW Hispanic/Mexican-American population database]* Please specify the value of theta that you use for the Combined Hispanic, SW Hispanic, or Mexican-American population database that you use in casework, and will use in this study (Note: negative values and values greater than 0.2 will be regarded as typographical errors): _____

5. Please indicate which Overall Combined population database you use in your casework, and will use in this study:
   - *5.a NIST 1036 Revised All (2017)*
   - *5.b Other (Please specify: _____)*
   - *5.c We do not use an Overall Combined population database*

5.1 *[if you use an Overall Combined population database]* Please specify the value of theta that you use for the Overall Combined population database that you use in casework, and will use in this study (Note: negative values and values greater than 0.2 will be regarded as typographical errors): _____

3 Comparison Packets: DNA Mixture Profiles and Reference Profiles

In the *ICSA Subtest* you will be assigned a total of 8 *Comparison Packets*. Each *Comparison Packet* includes one DNA mixture profile, one or more reference profiles, positive and negative controls, and allelic ladders. No case information will be provided. All DNA mixture profiles and reference profiles are electropherograms, provided to participants as HID files.

Each *Comparison Packet* includes one of more reference profiles:

- All comparison packets include 1 reference profile designated as "person of interest" (POI), which for the purposes of this study indicates an individual whose contribution to the mixture is in question (such as an alleged perpetrator).

- All comparison packets that are simulated sexual assault kits (SAKs) include 1 reference profile labeled "victim," which for the purposes of this study indicates the complainant in a sexual assault from whom simulated sexual assault kit samples are collected.
- Some comparison packets that are simulated SAKs may include 1 reference profile labeled "consensual partner," which for the purposes of this study indicates an individual known to have had consensual intimate contact with a victim of a sexual assault.
- Some non-SAK comparison packets may include 1 reference profile labeled "expected contributor," which for the purposes of this study indicates a known individual who is expected or assumed to be a contributor to a DNA mixture profile, such as the owner of an item or a member of a household.

You will have access to only one *Comparison Packet* at a time: to avoid the possibility of administrative errors or misunderstandings, you must submit your responses for a DNA mixture profile before downloading the next DNA mixture profile.

3.1 Amp/CE Settings

In order to represent the SOPs of as many participating laboratories as feasible, electropherograms were prepared using four combinations of "Amp/CE Settings" (which refers to a specific combination of amplification kit, amplification cycles, volume of amplification reaction, CE instrument, and injection time and voltage). The combinations of Amp/CE Settings that have been implemented were the four most commonly-used Amp/CE settings selected by registered participants,[*] and use the following abbreviations:

- "6C29" — Promega PowerPlex Fusion 6C, 29 amplification cycles, 25μL amplification volume, ABI 3500xl, 1.2kV, 24 seconds (equivalent to ABI 3500: 1.2kV, 15 seconds[†])
- "GF28" — Applied Biosystems GlobalFiler, 28 amplification cycles, 25μL amplification volume, ABI 3500xl, 1.2kV, 24 seconds (equivalent to ABI 3500: 1.2kV, 15 seconds)
- "GF29" — Applied Biosystems GlobalFiler, 29 amplification cycles, 25μL amplification volume, ABI 3500xl, 1.2kV, 24 seconds (equivalent to ABI 3500: 1.2kV, 15 seconds)
- "ID28" — Applied Biosystems AmpFLSTR Identifiler Plus, 28 amplification cycles, 15μL amplification volume, ABI 3500xl, 1.2kV, 12 seconds (equivalent to ABI 3500: 1.2kV, 7.5 seconds)

You will be assigned mixtures that were prepared using the Amp/CE setting option that you chose during *Mixture Configuration Selection*, prior to the *NoC Subtest*.

3.2 Preparation of DNA Mixture Profiles

The DNA used to create the mixture profiles for this study came from various sources, including buccal, blood, and tissue samples. There were no simulated/contrived profiles; all DNA profiles in this study are from real people. DNA samples were extracted prior to mixing.

Mixtures were quantified using ABI Quantifiler Trio on an ABI 7500 real-time PCR instrument. The mixture quantification results (including the total amount of DNA amplified, amount of male DNA, and degradation index) will be included with the mixtures in the *ICSA Subtest*.

Various volumes of DNA were pipetted into a single tube to make a large mixture stock. That stock was then aliquoted and amplified in each of the four amp kits (see "Amp/CE settings" above for amplification volumes and cycles). The ABI 9700 thermocycler was used for amplification, using the specific Amp/CE settings and other standard manufacturer recommended settings. The ABI 3500xl was used for capillary electrophoresis

[*] *Registered participants were contacted by email and were given a deadline of 23 August 2021 to indicate preferences for Amp/CE settings.*

[†] *Based upon discussions with ThermoFisher, we are proceeding with the assumption that injection for X seconds on an ABI 3500@1.2kV can be considered equivalent to 1.6X seconds on an ABI 3500xl@1.2kV.*

(CE), using injection time and voltage settings specified in the Amp/CE Settings (see above); settings for run time, run voltage, capillary length, polymer type, etc. use the default settings specified for each amp kit.

GeneMapper (v1.5; incorporated into ABI 3500xl) was used to create HID files. We are not providing PDFs (images) of the electropherograms because creating such PDFs implements decisions regarding the analytical threshold (AT) value and the utilization of stutter filters, and we want all such decisions to be made by the participants.

Every effort was made with respect to quality assurance in creating these mixtures. Note that in some cases there may be artifacts (such as pull-up) present, as may be found in ordinary casework — please review the controls provided.

3.3 Data Provided

In the ICSA Subtest you will be assigned a total of 8 *Comparison Packets*. You will have access to only one *Comparison Packet* at a time: to avoid the possibility of administrative errors or misunderstandings, you must submit your responses for a DNA mixture profile before downloading the next DNA mixture profile.

Each *Comparison Packet* is numbered (ICSA_001 through ICSA_999, shown as "ICSA_XXX" in the table below). Participants are not necessarily assigned the same packets, and the order of assignments varies among participants.

Each *Comparison Packet* is specific to the Amp/CE Settings previously selected by participants (shown as "YYYY" in the table below), using the abbreviations listed in *Section 3.1 (Amp/CE Settings)* [6C29, GF28, GF29, or ID28].

Each *Comparison Packet* includes 1 DNA mixture profile and 1 person of interest (POI) reference profile. *Comparison Packets* that are simulated sexual assault kits (SAKs) include 1 victim (VIC) reference profile, and may include 1 consensual partner (CON) reference profile. Non-SAK comparison packets may include 1 expected contributor (EXP) reference profile. Each mixture or reference profile includes positive and negative controls, and 2 allelic ladders.

Each *Comparison Packet* is contained in a Zip file, downloaded from the DNAmix 2021 website (https://dnamix.edgeaws.noblis.org/). The mixture and reference profiles are included in separate subdirectories as shown in the table below.

	Subdirectory	*Files*	
All packets	ICSA_XXX_YYYY/Mixture/	**ICSA_XXX_YYYY_Mixture.HID**	***DNA mixture profile***
		ICSA_XXX_YYYY_Ladder1-Mixture.HID	*Controls for DNA mixture profile*
		ICSA_XXX_YYYY_Ladder2-Mixture.HID	
		ICSA_XXX_YYYY_NEG-Mixture.HID	
		ICSA_XXX_YYYY_POS-Mixture.HID	
	ICSA_XXX_YYYY/POI/	**ICSA_XXX_YYYY_POI.HID**	***Person of interest (POI) reference profile***
		ICSA_XXX_YYYY_Ladder1-POI.HID	*Controls for POI reference profile*
		ICSA_XXX_YYYY_Ladder2-POI.HID	
		ICSA_XXX_YYYY_NEG-POI.HID	
		ICSA_XXX_YYYY_POS-POI.HID	
Some packets	ICSA_XXX_YYYY/VIC/	**ICSA_XXX_YYYY_ VIC.HID**	***Victim (VIC) reference profile***
		ICSA_XXX_YYYY_Ladder1-VIC.HID	*Controls for VIC reference profile*
		ICSA_XXX_YYYY_Ladder2-VIC.HID	
		ICSA_XXX_YYYY_NEG-VIC.HID	
		ICSA_XXX_YYYY_POS-VIC.HID	
	ICSA_XXX_YYYY/CON/	**ICSA_XXX_YYYY_ CON.HID**	***Consensual partner (CON) reference profile***
		ICSA_XXX_YYYY_Ladder1-CON.HID	*Controls for CON reference profile*
		ICSA_XXX_YYYY_Ladder2-CON.HID	
		ICSA_XXX_YYYY_NEG-CON.HID	
		ICSA_XXX_YYYY_POS-CON.HID	
	ICSA_XXX_YYYY/EXP/	**ICSA_XXX_YYYY_ EXP.HID**	***Expected contributor (EXP) reference profile***
		ICSA_XXX_YYYY_Ladder1-EXP.HID	*Controls for EXP reference profile*
		ICSA_XXX_YYYY_Ladder2-EXP.HID	
		ICSA_XXX_YYYY_NEG-EXP.HID	
		ICSA_XXX_YYYY_POS-EXP.HID	

In some *Comparison Packets*, the positive or negative controls were re-injected, in which case they are in a subdirectory (named POS or NEG) with the associated ladders.

4 Reporting Statistical Analysis Results

To ensure there is no ambiguity in the reported results, all statistical values shall be reported using scientific notation, using the format X.XXE+YY or X.XXE-YY.

- X.XX represents the statistical value, using three significant digits
- E represents an exponential constant of 10
- +/- represent the direction of the resulting exponent (i.e., positive for statistical values $\geq$ 1 and negative for statistical values < 1)
- YY represents the value of the resulting exponent, using two significant digits (e.g., 06 for an LR of 1,000,000)

Specific statistical values shall be reported as follows:

- For likelihood ratio (LR), report the result of the ratio (i.e., compute H_p/H_d and report the resulting value). For example, an LR of 1 million shall be reported as 1.00E+06. An LR of 1 shall be reported as 1.00E+00.
- For random match probability (RMP) and modified RMP (mRMP), report X (the "denominator") from the 1 in X result format. In other words, an RMP of 1 in 1 million shall be reported as 1.00E**+**06 (i.e., using a positive exponent).
- For combined probability of inclusion (CPI) and combined probability of exclusion (CPE), report X (the "denominator") from the 1 in X result format. In other words, a CPI of 1 in 1 million shall be reported as 1.00E**+**06 (i.e., using a positive exponent). Do not report the raw probability: a 0.000001% probability of inclusion should be transformed to 1 in 1 million and reported as 1.00E+06.

5 ICSA Subtest Questions

On the *DNAmix 2021 website*, you will be asked to answer the following questions for ***each*** of the 8 comparison packets that you are assigned in the *ICSA Subtest*. The *ICSA Subtest* is completed online; this information is provided here as a reference.

As a quality assurance measure, the website will display an image of the electropherogram for the first several loci in the DNA mixture profile for the assigned *Comparison Packet*. Please ensure that you are submitting your responses for the given mixture profile.

After downloading the packet, we recommend clicking HOME in the main menu bar (at the top of the DNAmix webpage) and returning to the ICSA Subtest screen only when you are ready to enter your responses. (Some users have timeout issues if this window is left open.)

For each comparison packet assessed in the ICSA Subtest, you will be asked to review and confirm your responses prior to submission. After submission, your responses are considered final and cannot be changed.

Packet Assignment Details

1. Please re-enter the Participant ID shown at the top of the page (Dxxxx):______

> *Note: this information will be used for quality assurance purposes only. The Participant ID (a 5 character alpha-numeric string starting with D) is located at the top right of the Interpretation, Comparison, and Statistical Analysis (ICSA) Page (right about the electropherogram preview image).*

2. Please double-check the Comparison Packet number: verify that the number of the HID mixture file and any reference files you are assessing is the same as shown at the top of this page. Please enter that Comparison Packet number here (for example, in the ICSA Beta Test, you would enter 999):______

> *The Comparison Packet number is located in the filename, in the electropherogram preview image (located at the top of the ICSA Subtest page of the DNAmix 2021 website), and embedded within the electropherogram data. You do not need to enter the "ICSA_" portion; please only enter the three digit Comparison Packet number.*

2.1 Please indicate which (if any) reference profiles other than the POI are included in this *Comparison Packet* (check all that apply; leave blank if none):

> *Comparison packets that include a victim reference profile are simulated SAK packets; these may also include a consensual partner reference profile.*
>
> *Non-SAK packets may include an expected contributor reference profile.*

2.1-a Victim (VIC)
2.1-b Consensual partner (CON)
2.1-c Expected contributor (EXP)

Suitability and Number of Contributors

Questions 3-13 from the *Number of Contributors (NoC) Subtest* will be repeated exactly here, with the addition of Question 9.2 below. Please refer to the *NoC Subtest Instructions* for details about how to respond to each question.

The *ICSA Subtest* includes the following additional question, which is not in the *NOC Subtest*:

9.2 *[if indicated a range or minimum NoC]* (Given that you would report a range or minimum number of contributors) What NoC value did you use as a basis for your conclusions/analyses reported for this Comparison Packet regarding the person of interest (POI) as a potential contributor to the mixture sample?

In other words, when conducting your comparisons and statistical analyses for this Comparison Packet what value did you assume for number of contributors to the mixture sample?

Please report all statistical analyses in Q18, 19, and 22 below (if applicable) based upon your response to this question.

9.2-a 1 contributor
9.2-b 2 contributors
9.2-c 3 contributors
9.2-d 4 contributors
9.2-e 5 contributors
9.2-f 6 contributors
9.2-g 7 contributors
9.2-h 8 contributors
9.2-i I based analyses with respect to the POI on a range of number of contributors (not a single NoC value)

Note: You will only move on to the Comparison and Statistical Analysis questions below if you indicate that the DNA mixture sample is suitable for comparison and/or statistical analysis.

Comparison Conclusion — General Case

14. What is your conclusion regarding the person of interest (POI) as a potential contributor to the mixture sample?

In other words, did you determine that the POI can be included or excluded as a potential contributor to this DNA mixture profile? Alternatively, were you unable to include or exclude the POI as a potential contributor, thus yielding an inconclusive determination? If your laboratory SOPs do not permit you to report categorical conclusions, please indicate as such.

14.a Included— the POI is considered a possible contributor (also known as consistent with, support for inclusion, cannot be excluded/eliminated)
14.b Inconclusive— the POI can neither be included nor excluded as a potential contributor (also known as uninformative)
14.c Excluded— the POI is NOT a possible contributor (also known as eliminated, support for exclusion)
14.d We do not use categorical conclusions

15. Was your conclusion regarding the POI as a potential contributor to the mixture based upon manual comparison, statistical analysis, or both?

In other words, how did you conduct your interpretation, comparison, and/or statistical analysis of THIS mixture sample with respect to the POI? If you did not compute any statistics and reported your conclusion based solely upon a review of the electropherograms (e.g., comparison of alleles, evaluation of peak heights, consideration of stutter or other artifacts, etc.), indicate manual comparison only. If you did not review/compare the electropherograms manually prior to computing a statistic (either manually or using a software tool), indicate statistical analysis only. If you manually reviewed/compared the electropherograms and computed a statistical value, indicate both.

15.a Manual comparison only [Go to Additional Comments]
15.b Statistical analysis only
15.c Both manual comparison and statistical analysis

Statistical Analysis — General Case

16. What type of statistical values are you reporting for this comparison packet?

In other words, please indicate which statistic you computed with respect to the POI in THIS comparison packet. You are permitted to use different statistics on different comparison packets; however, the same type of statistic must be used within a comparison packet.

16.a *Continuous likelihood ratio (LR)*
16.b *Semi-continuous LR*
16.c *Binary LR*
16.d *Combined probability of inclusion (CPI)*
16.e *Combined probability of exclusion (CPE)*
16.f *Random match probability (RMP)*
16.g *Modified RMP (mRMP)*

17. What software was used to calculate the statistical values you are reporting for this comparison packet?

In other words, please indicate which software you used to compute your statistics with respect to the POI in THIS comparison packet. You are permitted to use different software (or to use manual computation/in-house workbooks) on different comparison packets; however, the same software tool must be used to compute all statistics within a comparison packet.

17.a *I did my calculations manually, or used an in-house software or in-house workbook*
17.b *Armed Xpert*
17.c *CEESIt*
17.d *DNA View Mixture Solution*
17.e *EuroForMix*
17.f *LabRetriever*
17.g *likeLTD*
17.h *LRmix/LRmix Studio*
17.i *PopStats*
17.j *Soft Genetics MaSTR*
17.k *STRmix*
17.l *TrueAllele*

18. Statistical responses (General case). Please enter the result of your statistical analysis regarding the POI as a contributor to the mixture, for each of the following population databases.

Report your statistics using the type of statistics you indicated in Question 16, computed using the population database and theta you indicated in ICSA Settings Selection. If you do not use a given type of population database the corresponding question will be disabled.

*Report statistical values in scientific notation, using the format X.XXE+YY or X.XXE-YY.**

For likelihood ratios, use the following generalized propositions, assuming unrelated contributors: Hp = the POI is a contributor to the mixture; Hd = the POI is NOT a contributor to the mixture.

For LR values, report the point estimate of the computed LR (i.e., not an interval/range, a unified statistic, varNOC, stratified statistic, etc.)

18.1 *[if Black/African American DB]* Statistical value regarding the POI as a contributor to the mixture (Black/African American population database): _____

18.2 *[if White/Caucasian DB]* Statistical value regarding the POI as a contributor to the mixture (White/Caucasian population database): _____

18.3 *[if Combined Asian/ East Asian/Chinese DB]* Statistical value regarding the POI as a contributor to the mixture (Combined Asian/East Asian/Chinese population database): _____

18.4 *[if Combined Hispanic/SW Hispanic/Mexican-American DB]* Statistical value regarding the POI as a contributor to the mixture (Combined Hispanic/SW Hispanic/Mexican-American population database): _____

18.5 *[if Overall Combined DB]* Statistical value regarding the POI as a contributor to the mixture (Overall Combined population database): _____

* *Scientific notation format is detailed in Section 4.*

19. [if Amp/CE Setting Selection is 6C29, GF28, or GF29] Statistical responses (General case—CODIS core loci*). Please enter the result of your statistical analysis regarding the POI as a contributor to the mixture, for each of the following population databases, computed using only the 20 CODIS core loci. (Note: this question is optional)

> *Report your statistics using the type of statistics you indicated in Question 16, computed using the population database and theta you indicated in ICSA Settings Selection; use ONLY the 20 CODIS core loci in your computations. If you do not use a given type of population database the corresponding question will be disabled.*
>
> *Report statistical values in scientific notation, using the format X.XXE+YY or X.XXE-YY.*
>
> *For likelihood ratios, use the following generalized propositions, assuming unrelated contributors: Hp = the POI is a contributor to the mixture; Hd = the POI is NOT a contributor to the mixture.*
>
> *For LR values, report the point estimate of the computed LR (i.e., not an interval/range, a unified statistic, varNOC, stratified statistic, etc.)*

19.1 [if Black/African American DB] Statistical value regarding the POI as a contributor to the mixture based upon the 20 CODIS core loci (Black/African American population database): ______

19.2 [if White/Caucasian DB] Statistical value regarding the POI as a contributor to the mixture based upon the 20 CODIS core loci (White/Caucasian population database): ______

19.3 [if Combined Asian/ East Asian/Chinese DB] Statistical value regarding the POI as a contributor to the mixture based upon the 20 CODIS core loci (Combined Asian/East Asian/Chinese population database): ______

19.4 [if Combined Hispanic/SW Hispanic/Mexican-American DB] Statistical value regarding the POI as a contributor to the mixture based upon the 20 CODIS core loci (Combined Hispanic/SW Hispanic/Mexican-American population database): ______

19.5 [if Overall Combined DB] Statistical value regarding the POI as a contributor to the mixture based upon the 20 CODIS core loci (Overall Combined population database): ______

Comparison Conclusion — Sibling Case

20. In your casework, would you report a conclusion and/or statistics regarding the POI if a sibling (not an identical twin) of the POI is also a suspect, but for whom no DNA profile is available?

> *In other words, if a POI claims his sibling was the culprit but that sibling cannot be located for a sample, would you report a conclusion and/or statistical value regarding your interpretation, comparison, and statistical analysis of the mixture profile with respect to the POI?*

20.a *No* [Go to Q23, Conditioning]
20.b *Yes*

21. [if yes] What conclusion would you report regarding the POI if a sibling (not an identical twin) of the POI is also a suspect, but for whom no DNA profile is available?

> *In other words, did you determine that the POI can be included or excluded as a potential contributor to this DNA mixture profile (when considering that a sibling is also a suspect)? Alternatively, were you unable to include or exclude the POI as a potential contributor, thus yielding an inconclusive determination? If your laboratory SOPs do not permit you to report categorical conclusions, please indicate as such.*

21.a *Included— the POI is considered a possible contributor (also known as consistent with, support for inclusion, cannot be excluded/eliminated)*

---

* *A list of the 20 CODIS core loci can be found at:* https://www.fbi.gov/services/laboratory/biometric-analysis/codis/codis-and-ndis-fact-sheet

21.b Inconclusive— the POI can neither be included nor excluded as a potential contributor (also known as uninformative)
21.c Excluded— the POI is NOT a possible contributor (also known as eliminated, support for exclusion)
21.d We do not use categorical conclusions

Statistical Analysis — Sibling Case

22. Statistical responses (Sibling case). Please enter the result of your statistical analysis regarding the POI as a contributor to the mixture if a sibling (not an identical twin) of the POI is also a suspect (but for whom no DNA profile is available), for each of the following population databases.

> *Report your statistics using the type of statistics you indicated in Question 16, computed using the population database and theta you indicated in ICSA Settings Selection. If you do not use a given type of population database the corresponding question will be disabled.*
>
> *Report statistical values in scientific notation, using the format X.XXE+YY or X.XXE-YY.**
>
> *For likelihood ratios, use the following generalized propositions, assuming unrelated contributors: Hp = the POI is a contributor to the mixture; Hd = the sibling of the POI is a contributor to the mixture and the POI is NOT a contributor to the mixture.*
>
> *For LR values, report the point estimate of the computed LR (i.e., not an interval/range, a unified statistic, varNOC, stratified statistic, etc.)*

22.1 *[if Black/African American DB]* Statistical value regarding the POI as a contributor to the mixture given a sibling is also a suspect (Black/African American population database): ______

22.2 *[if White/Caucasian DB]* Statistical value regarding the POI as a contributor to the mixture given a sibling is also a suspect (White/Caucasian population database): ______

22.3 *[if Combined Asian/ East Asian/Chinese DB]* Statistical value regarding the POI as a contributor to the mixture given a sibling is also a suspect (Combined Asian/East Asian/Chinese population database): ______

22.4 *[if Combined Hispanic/SW Hispanic/Mexican-American DB]* Statistical value regarding the POI as a contributor to the mixture given a sibling is also a suspect (Combined Hispanic/SW Hispanic/Mexican-American population database): ______

22.5 *[if Overall Combined DB]* Statistical value regarding the POI as a contributor to the mixture given a sibling is also a suspect (Overall Combined population database): ______

Conditioning

23. *[If selected 2.1-a, 2.1-b, or 2.1-c]* Did you assume the presence of the victim, consensual partner, and/or expected contributor reference profiles during deconvolution? (check all that apply; leave blank if none apply)

> *In other words, did you assume that the victim and/or consensual partner (for SAK comparison packets) or the expected contributor (for non-SAK comparison packets) were contributors to the DNA mixture at any point during your interpretation and comparison of the mixture profile? Such assumptions may have been considered/incorporated either manually or using software.*

23.a I assumed the presence of the Victim (VIC)
23.b I assumed the presence of the Consensual partner (CON)
23.c I assumed the presence of the Expected contributor (EXP)

* *Scientific notation format is detailed in Section 4.*

24. *[If selected 2.1-a, 2.1-b, or 2.1-c]* Were your reported statistics conditioned on the victim and/or consensual partner OR on the expected contributor? (i.e., were these accounted for in the propositions, so that the statistics were calculated assuming their presence) (check all that apply; leave blank if none apply)

> *In other words, did you assume that the victim and/or consensual partner (for SAK comparison packets) or the expected contributor (for non-SAK comparison packets) were contributors to the DNA mixture during your statistical analysis of the mixture profile?*
>
> *Specifically, did your statistical analyses omit specific alleles/genotypes from your statistical analysis with respect to the POI based upon the genotypes of the victim, consensual partner, or expected contributor? Such omissions may have been incorporated in your statistical analysis conducted via manual computation or a software tool.*

24.a Statistics were conditioned on the presence of the Victim (VIC)
24.b Statistics were conditioned on the presence of the Consensual partner (CON)
24.c Statistics were conditioned on the presence of the Expected contributor (EXP)

Omitted Loci

25. Did you omit any loci from your statistical computations?

> *In other words, did you completely ignore any loci in your statistical computations, conducted either manually or using a software tool? These loci would be dropped from your computation entirely (i.e., you would be omitting ALL alleles at a given locus, not just a selection of alleles).*

25.a Yes
25.b No [Go to Additional Comments]

26. *[If Yes to omitted loci]* Please check all loci that you OMITTED from your statistical computations. (check all that apply; select at least one)

> *Note: you will only see the set of loci included in the amplification kit that you previously selected in Mixture Configuration Selection.*
>
> *Names of commercial manufacturers are included for the systems that are the most frequently used by registered participants; inclusion does not imply endorsement by the study team.*

Applied Biosystems GlobalFiler (display order)	Promega PowerPlex Fusion 6C (display order)	Applied Biosystems AmpFLSTR Identifiler Plus (display order)
D3S1358	Amel	D8S1179
vWA	D3S1358	D21S11
D165539	D1S1656	D7S820
CSF1PO	D2S441	CSF1PO
TPOX	D10S1248	D3S1358
Y indel	D13S317	TH01
Amel	Penta E	D13S317
D8S1179	D16S539	D16S539
D21S11	D18S51	D2S1338
D18551	D2S1138	D19S433
DYS391	CSF1PO	vWA
D2S441	Penta D	TPOX
D19S433	TH01	D18S51
TH01	vWA	Amel
FGA	D21S11	D5S818
D22S1045	D7S820	FGA
D5S818	D5S818	
D13S317	TPOX	
D7S820	D8S1179	
SE33	D12S391	
D10S1248	D19S433	
D1S1656	SE33	
D12S391	D22S1045	
D2S1338	DYS391	
	FGA	
	DYS576	
	DYS570	

Additional Comments

27. Additional comments: Please provide a comment ONLY if there is an issue or a limitation for this comparison packet that you could not adequately address using any of your responses above. (Please limit your responses to 75 words or less.)

DNAmix 2021 Informed Consent Form

Study Title:	Inter-laboratory Variation in Interpretation of DNA Mixtures<br>("DNAmix 2021")
Sponsors:	Noblis, Inc. and Bode Technology
Principal Investigators:	R. Austin Hicklin, Ph.D. (Noblis) (703) 610-1995, hicklin@noblis.org<br>Jonathan Davoren, M.S. (Bode) (703) 317-7400, jonathan.davoren@bodetech.com
Additional Contact:	Will Chapman, (703) 610-2983, william.chapman@noblis.org
Address:	Noblis, 2002 Edmund Halley Drive, Reston, VA 20191 USA

Purpose

This study will be a large-scale, independent, rigorous empirical evaluation of the extent of variation among forensic laboratories in the statistical analysis and interpretation of electropherograms (EPGs) resulting from DNA mixtures. We plan to have between 50 and 150 laboratories taking part in this study. This study is being conducted under a grant from the National Institute of Justice (NIJ Grant #2020-R2-CX-0049).

Participation

Participation will be open to all forensic laboratories that conduct DNA mixture interpretation as part of their SOPs; non-U.S. laboratories are welcome to participate if they report interpretations in English. Participation in the study requires the participants to agree to use the same diligence in performing these analyses as used operationally in casework, and to use their laboratory's SOPs in performing these analyses.

Procedures

The study will consist of four subtests:

1. *Policies and Procedures Questionnaire* — Online questionnaire to assess laboratory policies and standard operating procedures (SOPs) relevant to DNA mixture interpretation, interpretation or statistical software used, and parameter settings.
2. *Scenario Questionnaire* — Online questionnaire presenting a number of casework-derived scenarios (without DNA data), asking participants to assess how they would conduct analysis for each scenario.
3. *Number of Contributors Subtest (NoC)* — Assessment of suitability and number of contributors, given electropherogram data from DNA mixtures.
4. *Statistical Analysis and Interpretation Subtest* — Report of statistical results and categorical interpretations, given electropherogram data from DNA mixtures provided with DNA profiles of reference samples.

All of the subtests will be administered online. Participants are encouraged to participate in the early subtests even if they do not participate in the later subtests.

If your laboratory expresses interest or agrees to participate, it will be sent a link to a website that will collect the laboratory name, point of contact, and email address for the point of contact. After this consent form is submitted, a link will be sent to the Policies and Procedures Questionnaire. Once a subtest of the study is completed, a link to the next subtest in the above list will be sent.

Confidentiality

Results will be confidential. No information about your laboratory will be released. No personally identifiable information (PII) will be released and results will not be attributed to participants. The research results will be published, but anonymity of participants will be maintained and results will not be associated with specific participants. Personally identifiable information (PII) will be used only for the purpose of conducting the study, and will not be used or released for other purposes. Your laboratory's study results will not be linked to its PII. No reference will be made in oral or written reports,

publications, or released datasets that could link your laboratory's name to the study. A blind coding system will ensure anonymity. The subject ID numbers associated with your laboratory will be anonymized so that the analysis team will not be able to associate your laboratory's responses to any/all of the four subtests with your laboratory's name, email address, or laboratory representative. Cross-references between the subject IDs and the anonymized codes will be destroyed prior to the publication or public presentation of any results. Therefore, the identities of participating laboratories will not be associated with the results at any point during analysis, and such association will not be possible subsequently, such as for discovery.

Upon publication of the study results, your laboratory will be offered an opportunity to see its results using an Anonymous ID that will be provided when the last responses are submitted. This Anonymous ID will only be provided once: if your laboratory loses its Anonymous ID after it has been provided, your laboratory will not be able to see its results as the researchers will have no way of linking the Anonymous ID to your laboratory's name or contact information once the study is completed. The study team will have no way of knowing if your laboratory accessed its Anonymous ID. If your laboratory chooses to obtain its results, it is solely up to your laboratory's discretion as to whether or not to share its results with anyone, except as required by law.

The researchers will not disclose which laboratories did or did not take the test. In reporting results, results will be aggregated across multiple laboratories. Care will be taken so that the results are not aggregated in a way that compromises anonymity. The Principal Investigators and the Institutional Review Board (IRB) will be able to inspect confidential study-related records that identify your laboratory by name, which means that absolute confidentiality cannot be guaranteed.

Benefits

This study is for research purposes only. There is no direct benefit to your laboratory from participation in the study. The results of this study will be published in a peer-reviewed journal. The results of this research will provide the DNA analysis community with information regarding the accuracy, reproducibility, and repeatability of analyses produced in the discipline. This information can be used to improve analysis methodologies, training programs**,** and quality assurance measures. If the study indicates high performance by DNA analysis laboratories, this research may provide confidence in the legal community that DNA mixture analysis is reliable and provides added value to investigations and during courtroom proceedings. This research will inform future DNA mixture analysis studies.

Risks and Discomforts

No deception will be used in this study.

Analysts may experience physical fatigue (including eye strain) and mental fatigue if they perform analyses for an extended period of time. The scenarios in the Scenario Subtest are designed to resemble real casework and may be disturbing to some people.

In some legal systems, knowledge of your laboratory's test results may create an obligation for your laboratory to disclose those results in a criminal, civil or regulatory proceeding for which your laboratory is called to testify or provide evidence. If your laboratory chooses to access its own results, that information may then be under legal discovery when the laboratory acts as an expert witness in the future. If your laboratory elects to request its individual results, your laboratory is advised to consider first consulting with your laboratory's agency's counsel or counsel of your laboratory's choice.

The study team is not aware of any other risks, but there may be unknown risks associated with this study.

New Findings

Any new important information that is discovered during the study and which may influence your laboratory's willingness to continue participation in the study will be provided to your laboratory.

Alternatives

This research study is for research purposes only. Your laboratory is free to participate or not participate in this study. If your laboratory chooses not to participate, there will be no negative consequences.

Costs and Compensation for Participation

No charges will be billed to your laboratory or your agency for this study. Your laboratory will not be paid for its participation in this study.

Whom to Contact

If your laboratory has questions about the study, please contact the study staff listed on page one of this document. Please reference "DNAmix 2021" when contacting the Principal Investigators or study staff.

Refusal or Withdrawal of Participation

Participation in this study is voluntary. Participation in this research study is not mandatory; your laboratory may withdraw from the study for any reason without penalty. If your laboratory decides to participate, completion of the entire study is encouraged, but is not required. If your laboratory withdraws from the study before data collection is completed, you may notify the principal investigators if you wish for your laboratory's data to be destroyed. After the end of the data collection period (after the results from all participants have been collected), data will be anonymized and pooled and withdrawal of your laboratory's data will not be possible. Your laboratory's decision whether or not to participate in the study will not affect its current or future relations with the investigators. The investigators or the sponsor can stop your laboratory's participation at any time without your laboratory's consent.

Injury Statement

If anyone at your laboratory becomes ill or is injured while your laboratory is in the study, that individual is encouraged to get the medical care that s/he needs right away. If anyone at your laboratory is injured while engaged in the study or as a direct result of this study, your laboratory should contact the principal investigator at the number(s) provided on the first page of this form. Your laboratory will not lose any of its legal rights or release the sponsor, the Investigator, the study staff, or study site from liability for mistakes by signing this consent document.

Data Use Agreement

Due to human subjects research restrictions, the DNA profiles and mixtures included in this study shall not be used for any purpose other than this study: they shall not be stored, retained, or shared with anyone outside your laboratory; they shall not be used for research or internal validation studies; any copies or representations of the DNA profiles and mixtures shall be destroyed at the end of the study.

Consent to Take Part in this Research Study

I confirm that the purpose of the research, the study procedures and the possible risks and discomforts as well as potential benefits that I may experience have been explained to me. Alternatives to my laboratory's participation in the study also have been discussed. All of my questions have been answered. I have read this consent form. By agreeing to this informed consent form, my laboratory agrees that it will not save, copy, or redistribute any of the files included in the study.

Laboratory Name: ______________________________

Laboratory Representative's Name: ______________________________

By selecting "I consent to taking part in this study" below, as an authorized representative of my laboratory, I freely give consent for my laboratory to take part in this study.

Please select one:

☐ *I consent to taking part in this study.*

☐ *I do NOT consent to taking part in this study.*

DNAmix 2021 Mixture Analysis Study

Frequently Asked Questions

Last updated 11 April 2022

See the end for the most recent additions

Note: All questions received over the course of the study will be answered here as well as in direct email responses, unless the questions are specific to an individual participant. This allows us to provide the same information to all participants. We may modify the wording of some questions we receive to remove lab-specific references, or to combine multiple similar questions.

---

1. ***Q: Who may participate?***

   A: Participation will be open to all forensic laboratories that conduct DNA mixture interpretation as part of their SOPs; non-U.S. laboratories are welcome to participate if they report interpretations in English. Participation in the study requires the participants to agree to use the same diligence in performing these analyses as used operationally in casework, and to use their laboratory's SOPs in performing these analyses.

2. ***Q: Are you looking for only a single response per lab, or could multiple analysts within my lab participate?*** (added 30 June)

   A: The responses must go through technical review/quality assurance as specified by the laboratory's SOPs. As long as that is satisfied, multiple responses from a given laboratory are acceptable. We refer to multiple participants from a lab as "subunits" (as discussed in the instructions).

3. ***Q: For the NoC and ICSA subtests, will data be provided from only a single amplification kit or the amplification kit which is actually used by the participant laboratories?*** (added 30 June)

   A: We will prepare HID files for several popular combinations of amplification kit, CE instrument, and amplification cycle (collectively "Amp/CE Settings"). Specifics will be determined by the results of the Configuration Questionnaire completed as part of registration.

4. ***Q: Is a cell phone really necessary for registration? I do not have a work cell, and am hesitant to use my personal cell for this.*** (added 30 June)

   A: Unfortunately, we do require a cell phone number for registration. Security approval for the website requires multi-factor authentication by texting a code to a cell phone — this level of security is required to protect both the participants' information, and the DNA profiles included in the study. The cell phone number will only be used for that purpose, will never leave the server, and is not accessible by the study staff. If this is a burden, let us know: we are looking into other alternatives, but at least for now this is our only option.

5. ***Q: I had a question regarding the subunits. All of the qualified analysts in my section would like to participate in the study, so I was considering splitting up into groups of two, with one completing the analysis/interpretation and the other completing the technical review. Would it be reasonable to split up each phase (the NoC subtest and the ICSA subtest) between the two analysts? Could I allow half of the NoC subtest to be completed by analyst 1, have it tech reviewed by analyst 2, and then allow analyst 2 to complete the second half of the subtest and have analyst 1 tech review? I want to allow as much participation in the study as possible while still having everything technically reviewed, if possible.*** (added 30 June)

   A: The simple answer: How you assign staff to perform the subtests is your laboratory's decision. More broadly, with context: From a human subjects research standpoint, the participants in this study are laboratories (or

subunits within laboratories) — not individuals. The study team does not need (or want) visibility into whether a participating subunit is an individual or team: we only ask that the labs do what they can to make this as close to casework as possible, including tech review, and that the responses from subunits within a lab be independent.

6. *Q: Will all 4 studies be opened to a subunit even if that individual misses a particular study? For example, if an individual signs up as a subunit but misses study 1, will subsequent studies then become unavailable to that individual?* (added 30 June)

   A: We encourage participants to complete all 4 phases of the study, and they will be made available in order. Since the 1st 2 phases (the P&P Questionnaire and Casework Scenario (CS) Questionnaire) are just questionnaires, we assume that participants will complete those prior to the last 2 phases (NoC Subtest and ICSA Subtest): our analyses of the NoC and ICSA responses will rely on the P&P and CS responses.

7. *Q: What is the study schedule? What is the timeframe for participating labs to return results?* (updated 11 April)

   A: Our current planned schedule is as follows:
   - *Registration will be open for new participants through 06 Mar 2022.*
   - *P&P results will be accepted through 10 Mar 2022.*
   - *Casework Scenarios results will be accepted through 13 Mar 2022.*
   - *NoC results will be accepted through 19 Apr 2022.*
   - *ICSA results will be accepted through 05 June 2022.*

8. *Q: During Registration, there is a spot on the Informed Consent Form to add the "Laboratory Name" and "Laboratory Representative's Name," to give consent to take part in the study. Should the name be one of the individuals in the subunit, or just one representative per laboratory?* (added 7 July)

   A: On the Consent Form, the Laboratory Representative is an authorized representative of the lab: it is the lab's discretion who that is, and could be the point of contact, a manager, lab director, or legal counsel. If multiple subunits from a lab are participating, it is the lab's discretion whether there is a single lab representative on the Consent Form or a different one for each subunit. The name of the lab representative from the Consent form does not have to be the point of contact from the Registration form (but it can be). (The point of contact does need to be different for each subunit.)

9. *Q: If a laboratory opts to have teams of multiple analysts complete the study, for ease & consistency, can one team fill out the study questionnaires for the whole laboratory/unit?* (added 7 July)

   A: We prefer to treat the subunits as independent participants as much as possible, and request that each subunit complete the questionnaires separately.

10. *Q: If the laboratory opts to have teams and one team was unable to hand in their results, would that void the rest of the participants participation from the study?* (added 7 July)

    A: No: we will treat the responses from the subunits independently. Note also that we use partial results from participants who do not complete the entire study (e.g. if a lab responds to the questionnaires and NoC subtest but does not finish the ICSA subtest)

11. *Q: When will the closing period for registration be for this study?* (added 7 July)

    A: We will keep registration open at least through mid-September (to accommodate potential participants who hear of the study at ISHI).

12. *Q: Could you give me an idea of the time it should take to participate in the project?* (added 29 July)

    A: We estimate the time needed for participants for each sample in the Number of Contributors (NoC) Subtest to vary significantly based on the complexity of the mixture, from 5 minutes to over an hour per sample. We estimate the time needed for participants for each sample in the Interpretation, Comparison, and Statistical Analysis (ICSA)

Subtest to vary significantly, up to 3 or 4 hours of human analyst time per sample, not including software processing time.

13. *Q: (Regarding the Policies and Procedures question "Do your SOPs limit interpretation and/or comparison based on a maximum total number of contributors?") My laboratory has different analysis paths depending upon sample/case conditions, each with different limitations on maximum total number of contributors. How should I record my response?* (added 05 August, modified 21 September)

    *A: We recognize that laboratories may have different interpretation/comparison/statistical analysis paths depending upon the case or sample conditions. Given this, please record the single highest possible **total** NoC that your laboratory has the ability to handle for DNA mixture samples.*

14. *Q: In the Casework Scenarios Questionnaire (Phase 2), you ask in question 2 which types of information we USUALLY have available during interpretation of DNA data. When you refer to "Sexual Assault Medical Exam Report," what specifically does this include?* (added 21 September)

    A: The Sexual Assault Medical Exam Report will include details regarding the source of specific samples (e.g., vaginal swab, rectal swab, etc.), the presence of bruises or injuries (e.g., descriptions, photographs, etc.), the medical condition of the victim, and/or whether the victim had consensual sex prior to the assault.

15. *Q: In the Casework Scenarios Questionnaire (Phase 2), you ask in question 2 which types of information we USUALLY have available during interpretation of DNA data. While my laboratory does not have formal statements from witnesses, complainants, or investigators, we do have some information provided to use regarding the case by means of a case history on our submission forms. Can you please clarify how you would like me to indicate as such in question 2.* (added 21 September)

    A: Your description of the case history provided on submission forms would be included in the "Law Enforcement Case File." Please check this box if you USUALLY have access to that case history information.

16. *Q: I was disappointed that the Qiagen Investigator 24plex kit was not included as one of the subset whilst two GlobalFiler settings were chosen instead. Why was this kit not included in the study?* (added 21 September)

    A: We wish we could replicate all the registered participants STR laboratory protocols, but are unfortunately unable to provide all possible Amp/CE settings due to funding and time constraints. In selecting the specific Amp/CE settings options that we would provide, we used the four most commonly-used combinations of settings (as reported by registered participants by 23-Aug). Overall, there was a clear majority of participants who used GlobalFiler / 29 cycles or Fusion 6C / 29cycles, but the remainder of participants had a wide variety of settings. Relatively few participants selected Qiagen Investigator 24plex and those who did had a wide variety of cycle settings, so there was no single set of 24plex Amp/CE settings that would have captured a statistically-useable number of potential participants.

17. *Q: Since we're a laboratory using 24plex with STRmix, since our model maker is not adjusted for any of the kits, will it still make sense for us to move forward (i.e. will model maker for one of the sets be included, SOPS, etc.)?* (added 21 September)

    A: For each Amp/CE setting combination, we will be providing the following data/information:
    - *1 DNA mixture profile (HID file)*
    - *0-3 DNA reference profiles (HID files)*
    - *The following textual information:*
        - *Amp/CE Settings used to create the electropherograms:*
            - *Amplification kit*
            - *Amplification cycles*
            - *Volume of amplification reaction*
            - *CE instrument*

    - *Injection time and voltage*
  - *Quantitation data (as measured by Quantifilier Trio during quantitation of the mixture):*
    - *Total amount of DNA amplified*
    - *Total amount of male DNA amplified*
    - *Degradation index*
- *The following additional HID files, which some participants may wish to use for quality assurance:*
  - *Amplification positive control (HID file)*
  - *Amplification negative control (HID file)*
  - *2 allelic ladders (2 HID files)*

We will not be providing the model maker or SOPs for each Amp/CE setting combination. It is up to the participating laboratory to use the resources available to them.

Given this, it is up to the discretion of the laboratory whether they choose to move forward to NoC and ICSA using an Amp/CE Settings option that differs from their SOPs. You will have an opportunity to indicate whether or not you choose to move forward in the forthcoming Mixture Configuration Selection. Should you choose to participate in NoC and/or ICSA using Amp/CE Settings that are not equivalent to your SOPs, you will have the opportunity to indicate as such.

Even if you are unable to participate in NoC and ICSA, we encourage laboratories to participate in the first two phases of the study (Policies & Procedures Questionnaire and Casework Scenarios Questionnaire). Please let us know if you have any further questions.

18. *Q: I am attempting to submit my responses to the NoC Subtest, but when I click "Submit" after reviewing, I get this error message: "An error occurred while submitting responses."* (added 22 November)

A: This error is a result of a browser, network, or timeout issue. To fix this issue, please logout of the DNAmix webpage and close your browser; then reopen a new browser window and log back into the DNAmix webpage. Once you have logged back in, you can re-renter and review your responses and you should be able to successfully submit.

To prevent this issue from occurring for future submissions: After downloading the packet, we recommend clicking HOME in the main menu bar (at the top of the DNAmix webpage) and returning to the NoC Subtest screen only when you are ready to enter your responses. (Some users have timeout issues if this window is left open.)

19. *Q: What changes were made after the ICSA Beta Test?* (added 19 January)

A: Two changes were made since the ICSA Beta Test:

- *Responses entered into the website are now automatically saved: if you enter part of your responses for a sample and close the browser, the responses are retained the next time you log into the website.*
- *We added one question (#9.2) to address participants who would report the number of contributors (NoC) as a range (or minimum), but would use a single NoC value when calculating statistics with respect to a person of interest.*

20. *Q: There are no Amp/CE choices for the 3130 genetic analyzer; therefore, we do not have comparable peak heights when referring to our analytical and stochastic thresholds. Do you have an alternative solution or should we consider ourselves unable to participate?* (added 21 January)

A: We wish we could replicate all the registered participants STR laboratory protocols, but are unfortunately unable to provide all possible Amp/CE settings due to funding and time constraints. In selecting the CE instrument options that we could provide, we evaluated the responses to the Registration Questionnaire (as completed by registered participants by 09-Aug-2021), which indicated a supermajority of participants used the ABI 3500 or 3500xl genetic analyzer. Given this, we opted to move forward using only the ABI 3500 series instruments to prepare the samples in this study, in order to maximize the number of different Amp/CE settings we could accommodate. We will not be providing data for any other genetic analyzers.

Given this, it is up to the discretion of your laboratory whether you choose to move forward to NoC and ICSA using an Amp/CE Settings option that differs from their SOPs in terms of the CE instrument used. Should you choose to participate in NoC and/or ICSA using Amp/CE Settings that are not equivalent to your SOPs, you will have the opportunity to indicate as such.

Even if you are unable to participate in NoC and ICSA, we encourage laboratories to participate in the first two phases of the study (Policies & Procedures Questionnaire and Casework Scenarios Questionnaire).

21. *Q: Should we complete the NoC Subtest (Phase 3) if we are not able to complete the ICSA Subtest (Phase 4) due to time constraints because of our current laboratory initiatives/caseload?* (added 21 January)

A: Even if you are unable to complete the ICSA Subtest due to time constraints, we encourage laboratories to complete the first three phases of the study (Policies and Procedures Questionnaire, Casework Scenarios Questionnaire, and NoC Subtest). We are also willing to accept any number of ICSA Comparison Packets that you are able to complete in the study period (even if you are unable to complete all 8 assignments).

22. *Q: If multiple individuals from the same lab participate, each individual is asked to register as a "subunit" from a single laboratory. Will each "subunit" receive different mixtures for the NoC and ICSA subtests, or will they be given the same mixtures because they are linked to a single laboratory/point of contact?* (added 27 January)

A: Different participants will not necessarily receive the same mixtures as each other (in NOC or in ICSA). Labs with multiple participants, please note that the participants' responses must be independent: the participants may not collaborate with each other. The point of contact needs to be different for each subunit. (see FAQ#8 above)

23. *Q: Are we expected to report sub-source or sub-sub source likelihood ratios in the ICSA Subtest?* (added 28 January)

A: In the ICSA Subtest instructions, we indicate that "For LR values, report that point estimate of the computed LR (i.e., not an interval/range, a unified statistic, varNOC, stratified statistic, etc." More specifically, we are expecting laboratories to report LRs computed for the ***sub-source level proposition*** *(not the sub-sub-source level).*

This follows the best practices recommended by ASB Standard 041 (Formulating Propositions for Likelihood Ratios in Forensic DNA Interpretations, Draft 2021): "Within the capabilities of the analysis approach used, the laboratory should report results for a pair of propositions that addresses the issue of interest. This level is the highest level in the hierarchy for which the forensic scientist can provide information. Hence, the laboratory should report results given sub-source level rather than sub-sub-source level propositions."

For context, ASB 041 provides the following summary of the hierarchy of propositions: "Proposition pairs are classified by the level of information required to assist the trier of fact: offense (e.g., "Mr. X raped V"), activity (e.g., "Mr. X had intercourse with V"), source (e.g., "The semen came from Mr. X"), sub-source (e.g., "Mr. X is a contributor to this DNA"), and sub-sub-source (e.g., "Mr. X is the minor contributor to this DNA mixture")."

24. *Q: I do not see BP Sentry (the probabilistic genotyping software that my lab and several other U.S. labs use) listed as an option in the ICSA Subtest. Can you please add it to the list of software available?* (added 01 February)

A: We will add BP Sentry as a response option to Question #17 in the ICSA Subtest, but will not be able to update the website until Weds Feb 2. (We will also add an "Other commercial software not listed above" option for any additional commercial software tools that may be missing.)

Note that the response options provided in the ICSA Subtest were based upon these sources:

- *The initial responses were developed in multiple detailed discussions with the DNAmix Working Group, which contains a cross-section of notable experts from across the forensic DNA community*

- *We added additional options indicated by registered participants in the Policies and Procedures Questionnaire as of 13 Jan 2022*
- *We added additional options based on any feedback received from participants who completed the ICSA Beta Test as of 19 Jan 2022*

25. *Q: In the P&P Questionnaire (Phase 1), I am unable to enter my lab's default stochastic threshold value of 1250 RFU. Do you know how to get past the "Enter an integer" error that pops up?* (added 01 February)

A: There was an upper limit of 1000 RFU inadvertently set for this question that has now been removed.
**If anyone has had this issue when entering default values of your lab's analytical threshold (AT) or stochastic threshold (ST) in the P&P Questionnaire, please contact us so we can update your responses accordingly.*

26. *Q: I receive an error when unzipping ICSA packets containing reference profiles for consensual partners (CON). Can you please help me to access these files?* (added 24 February)

A: The name of the subfolder ("CON") refers to a system action or device reference in Windows, which causes unexpected compatibility issues when trying to extract the folder from the ZIP files.

Due to this Windows compatibility issue, all folders previously named "CON" were updated to "CONS." Please note that all study documents and packet contents descriptors will still use the "CON" abbreviation; only the subfolder names were updated.

27. *Q: The consensual partner in my ICSA packet is labeled "EC" in the HID file instead of "CON". Do I have the right file?* (added 28 February)

A: In short, "EC" stands for "Expected Contributor", but you can assume that in this ICSA Comparison Packet the "expected contributor" (EC) is the victim's consensual partner (CON).

For additional background:

- *The initial version of nomenclature for the study grouped all "elimination-type" reference profiles together (this included any victim, consensual partner, or expected contributor).*
- *Based upon discussion with the DNAmix Working Group, we decided to split each of these types of reference profiles out explicitly, as detailed in the instructions (and summarized below). We made this decision to improve clarity.*
    - *Victim (VIC)*
    - *Consensual partner (CON)*
    - *Expected contributor (EXP)*
- *Any HID files that contain "EC" in the header were likely created prior to this change.*
- *Please assume that "EC" corresponds to the type of reference profile indicated in the packet description and the subfolder from which the HID file originated (i.e., VIC, CON, or EXP).*

28. *My laboratory always reports a general LR for cases in which a sibling of the POI is also a suspect (but for whom no DNA is available). While our software does have a specific option to compute an updated LR for the sibling case, we generally do not report it unless specifically requested. Which values would you like us to report for the sibling case questions in ICSA?* (added 03 March)

*A: If your SOPs **ever** allow you to report the sibling case (wherein Hd = a sibling of the POI is a contributor to the mixture), then please provide those LR values here.*

29. *Can you send me a copy of my responses after I submit them?* (added 18 March)

A: Due to human subjects and study restrictions, we are not permitted to release individual study results to anyone during the study, including participants. Confidentiality restrictions require that any results we release are aggregated in such a way that individual participants cannot be identified.

However, you have two options if you would like a copy of your responses:

- *After review but prior to submission, you are more than welcome to screenshot your responses and print/save them for your records.*
- *If you complete all four phases of the study, you will be provided with the opportunity to receive your AnonID. Your AnonID will allow you to review your responses once the study is published.*
    - *Note: results for the NoC and ICSA Subtests will be released after the study close, but we cannot release any responses to the P&P Questionnaire or other responses that could compromise confidentiality.*
